# (Meta)genomic insights into the pathogenome of *Cellulosimicrobium cellulans*

**DOI:** 10.1038/srep25527

**Published:** 2016-05-06

**Authors:** Anukriti Sharma, Jack A. Gilbert, Rup Lal

**Affiliations:** 1 Department of Zoology, University of Delhi, Delhi, India; 2 Biosciences Division (BIO), Argonne National Laboratory, 9700 South Cass Avenue, Argonne, IL, USA; 3 Department of Surgery, University of Chicago, 5841 S Maryland Ave, Chicago, IL, USA; 4 Marine Biological Laboratory, Woods Hole, MA, USA

## Abstract

Despite having serious clinical manifestations, *Cellulosimicrobium cellulans* remain under-reported with only three genome sequences available at the time of writing. Genome sequences of *C. cellulans* LMG16121, *C. cellulans* J36 and *Cellulosimicrobium* sp. strain MM were used to determine distribution of pathogenicity islands (PAIs) across *C. cellulans,* which revealed 49 potential marker genes with known association to human infections, e.g. Fic and VbhA toxin-antitoxin system. Oligonucleotide composition-based analysis of orthologous proteins (n = 791) across three genomes revealed significant negative correlation (*P* < 0.05) between frequency of optimal codons (*F*_*opt*_) and gene G+C content, highlighting the G+C-biased gene conversion (gBGC) effect across *Cellulosimicrobium* strains. Bayesian molecular-clock analysis performed on three virulent PAI proteins (Fic; D-alanyl-D-alanine-carboxypeptidase; transposase) dated the divergence event at 300 million years ago from the most common recent ancestor. Synteny-based annotation of hypothetical proteins highlighted gene transfers from non-pathogenic bacteria as a key factor in the evolution of PAIs. Additonally, deciphering the metagenomic islands using strain MM’s genome with environmental data from the site of isolation (hot-spring biofilm) revealed (an)aerobic respiration as population segregation factor across the *in situ* cohorts. Using reference genomes and metagenomic data, our results highlight the emergence and evolution of PAIs in the genus *Cellulosimicrobium*.

With only three published species, *C. cellulans*^1^, *C. funkie*^2^, *C. terreum*^3^, and 31 16S rRNA gene sequences, the genus *Cellulosimicrobium* remains underrepresented in present NCBI reference databases. First proposed by Schumann *et al.*^1^, this genus has stayed taxonomically confounding with multiple reclassifications from the genera *Cellulomonas, Oerskovia, Brevibacterium* and *Arthrobacter*^1^. The ecological distribution of *Cellulosimicrobium* strains has been by and large limited to the mesophilic environments such as soil, marine sponges, and clinical materials. However, only two instances of isolation from extreme environments have been reported, including hot springs^4^ and Antarctic snow^5^. Currently, there are four sequenced genomes for the genus *Cellulosimicrobium*, including *Cellulosimicrobium* sp. strain MM^4^, *C. cellulans* LMG 16121 (NZ_CAOI00000000.1), *C. cellulans* J36 (NZ_JAGJ00000000.1), and *C. funkei* U11 (NZ_JNBQ00000000.1), which were isolated from biofilms (surface temperature >  57 °C) at the Manikaran hot springs (surface water temperature >  95 °C), aluminium hydroxide gel antacid, compost, and agricultural soil, respectively. The genus *Cellulosimicrobium* is associated with human infections such as meningitis, endocarditis, bacteremia, soft tissue infection, endophthalmitis, septic arthritis, and prosthetic joint infections^6^. Here we perform a detailed genome wide investigation using two available *C. cellulans* genomes i.e. LMG 16121 and J36 along with *Cellulosimicrobium* sp. strain MM (for its > 97% whole genome proximity to *C. cellulans* strains) to determine evolutionary processes that have shaped pathogenicity across the species *C. cellulans*.

Previous genomic studies of emerging pathogenic bacteria have revealed that pathogenicity islands (PAIs) contribute significantly towards organismal evolution by expression of infection-related factors^7^. Using the three draft genome sequences and a metagenomically-derived minimal genome, we have deciphered the pathogenic gene complements of species *C. cellulans*, which is attributed to 80% incidences of the total human infections for this genus^6^. Highlighting the close proximity of the functionally coupled ORFs, Fic and VbhA, the results provide the evidence for the ‘*selfish operon*’ theory whereby their juxtaposition on a PAI results from a probable single horizontal gene transfer event^8^. PAIs ORF annotation revealed 49 virulence-encoding genes, and suggested that horizontal transfer from non-pathogenic bacteria plays a signficiant role in the evolution of PAIs. Finally, these analyses provide a platform for using these 49 credible virulence markers to diagnose the presence of *Cellulosimicrobium* pathogens.

## Results and Discussion

### Phylogenomic analysis

**Figure 1:**
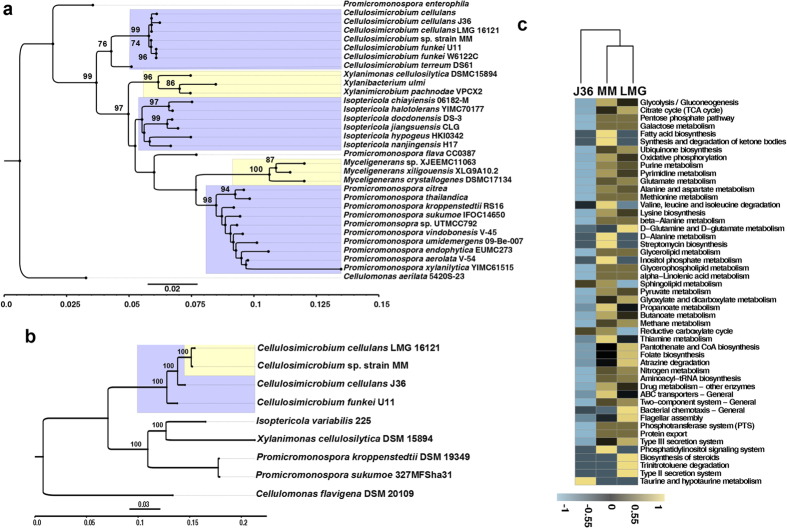
Phylogenomic analysis and comparative functional potential of *Cellulosimicrobium* strains. **(a)** Rooted Maximum likelihood tree based on Jukes-Cantor model for family *Promicromonosporacae* using 31 16S rRNA gene sequences with *Cellulomonas aerilata* 5420S-23 as outgroup, **(b)** Rooted tree based on 31 single copy genes from 7 whole genomes using *Cellulosmonas flavigena* DSM 20109 as outgroup. All the trees are drawn to scale, with branch lengths measured in the number of substitutions per site. The percentage (> 70%) of replicate trees in which the associated taxa clustered together in the bootstrap test (1000 replicates) are shown next to the branches. **(c)** Heatmap with column dendrogram showing top 50 metabolic pathways reconstructed between three *Cellulosimicrobium* genomes i.e. strains MM, LMG16121, and J36. Three strains were clustered based on functional pathways using Manhattan distance metric, top 50 pathways with standard deviation 0.4 and having at least 0.8% of the total abundance were selected. Colour scale is representing the relative abundance of each functional pathway.

Very few genomes or 16S rRNA sequences exist for *Cellulosimicrobium*, therefore, phylogenetic reconstruction for the strains was performed using the Family *Promicromonosporacae*, which significantly increased the number of genomes and 16S rRNA sequences available. 16S rRNA gene (n =  31) based tree topology revealed genus-specific clustering for *Promicromonospora* (n =  11), *Isoptericola* (n =  6), *Myceligenerans* (n =  3), *Xylanibacterium* (n =  1), *Xylanimonas* (n =  1), *Xylanimicrobium* (n =  1) and *Cellulosimicrobium* (n =  7)*. Cellulosimicrobium* sp. strain MM was clustered with *C. cellulans* LMG 16121 (Fig. 1a). Pairwise average nucleotide (ANI) values were also calculated generating a score of 98.23% (>95%) between Cellulosimicrobium needs to be italicized. sp. MM and *C. cellulans* LMG 16121, which suggested that the genome was a sub-species^9^. ANI values for *C. cellulans* J36, *C. funkei* U11, *I. variabilis* 225, *P. kroppenstedtii* DSM19349, *P. sukumoe* 327MFSha31, and *X. cellulosilytica* DSM 15894 with respect to strain MM, were 88.24%, 85.29%, 81.17%, 80.28%, 79.71%, and 79.88%, respectively, distinctly demonstrating species/genus level delineation (see Supplementary Table S2). Further, DNA-DNA hybridization (DDH) values were also determined in order to resolve strain MM at species level. % DDH values were 75.6, 55.3, 56, 20.9, 15.7, 16, 17.2 for *C. cellulans* LMG16121, *C. cellulans* J36, *C. funkei* U11, *I. variabilis* 225, *P. kroppenstedtii* DSM19349, *P. sukumoe* 327MFSha31, and *X. cellulosilytica* DSM 15894, respectively (see Supplementary Table S3). Both ANI and DDH values assigned *Cellulosimicrobium* sp. strain MM under *C. cellulans* species with values greater than species delination cut-off i.e. 95% and 70%, respectively for each analysis^9^. Interestingly, *C. cellulans* J36 demonstrated values less than the species delineation cut-off for both ANI and DDH which here indicates that this strain needs further confirmation using biochemical and physiological tests, to be put under *C. cellulans* species. Overall, the 16S rRNA analysis identified that *C. funkei* and *C. cellulans* form a clade, with *C. terreum* as an outlier to this clade (Fig. 1a). Multiple strains of *C. cellulans* were scattered into two sub-clades with *C. funkei* possibly because of the low number of *Cellulosimicrobium* strains with an available 16S rRNA sequence (n =  6; Fig. 1a). Whole genome based (n =  8) phylogenetic reconstruction using both 31 single copy genes^10^ (Fig. 1b) and 400 conserved bacterial marker genes^11^ (see Supplementary Fig. S1) revealed similar tree topology whereby strain MM was grouped with *C. cellulans* LMG16121.

### Comparative functional potential of *Cellulosimicrobium cellulans* strains

Metabolic pathway reconstruction for the three *Cellulosimicrobium* strains based on KAAS^12^, revealed a conserved set of central pathways like glycolysis/gluconeogenesis, TCA cycle, β -alanine metabolism, inositol phosphate metabolism, propanoate metabolism, and two-component system (TCS). *Cellulosimicrobium* sp. strain MM exhibited the pathways for fatty acid metabolism, synthesis and degradation of ketone bodies, and D-alanine metabolism, which were not present in the other two neighbors (Fig. 1c). D-alanine is proposed to be involved in biofilm production, adhesion and pathogenesis. In addition, synthesis and degradation of ketone bodies in strain MM appear to play a pivotal role in quorum sensing, which is also associated with biofilm formation^13^. LMG16121 uniquely encoded a Type-II secretion system (Fig. 1c) in contrast to the other strains. Out of 102 pathways reconstructed across all the strains, taurine and hypotaurine metabolism, which are involved in membrane stabilization, glycolysis and glycogenesis, were unique to *C. cellulans* J36 (Fig. 1c)^14^. Further, hierarchical clustering of the three genomes on the bases of top 50 enriched metabolic pathways, revealed closeness of strain MM and LMG16121, as also shown using phylogenetic analysis (Fig. 1b,c). In total, 791 orthologous genes were identified across the three genomes (see Supplementary Table S4), with the majority (n =  598) assigned to translation, metabolism and structure maintenance^15^.

Horizontal gene transfer (HGTs) candidates were determined across three *Cellulosimicrobium* strains. Strain LMG had the greatest number of potentially transferred genes (n =  367), followed by strain MM (n =  348) and J36 (n =  280) (see Supplementary Table S5). It is possible that the high number of horizontal transfer events in strains LMG16121 and MM is indicative of the extreme environment from which they were isolated, i.e. antacid and arsenic contaminated hot spring microbial mat, respectively. Another explaination for occurrence of frequent HGT events overall in all three genomes can be presence of mobile genetic elements and PAIs across *C. cellulans* genomes (as discussed in the section below), as mobile genetic elements facilitate HGTs^16^. Strain MM encoded 2-oxo-acid dehydrogenase, histidine kinase, FNR transcriptional regulator, FAD dependent oxidoreductase, Clp subunits, and hemin transport proteins on HGT loci. Whereas LMG16121 HGT loci included cobyrinic acid ac-diamide synthase, TetR family transcription regulator, chitin binding protein, luxR, copper oxidase, bleomycin resistance protein, and CheY proteins. The HGT loci for J36 revealed integrase, IS3/IS911 family transposase, daunorubicin resistance protein, and CLG chitinase B (see Supplementary Table S5). Additionally, hierarchical clustering was performed on annotated HGT candidates across three genomes i.e. strain MM, LMG16121 and J36 along with heatmap showing relative abundance of HGT genes. Strain MM was clustered with J36 based on the annotation of the HGT genes which is interesting here as strain MM coordinated with LMG16121 in terms of frequency of HGT events (see Supplementary Fig. S2).

### Evidence for G+C biased gene conversions across the genus *Cellulosimicrobium*

**Figure 2:**
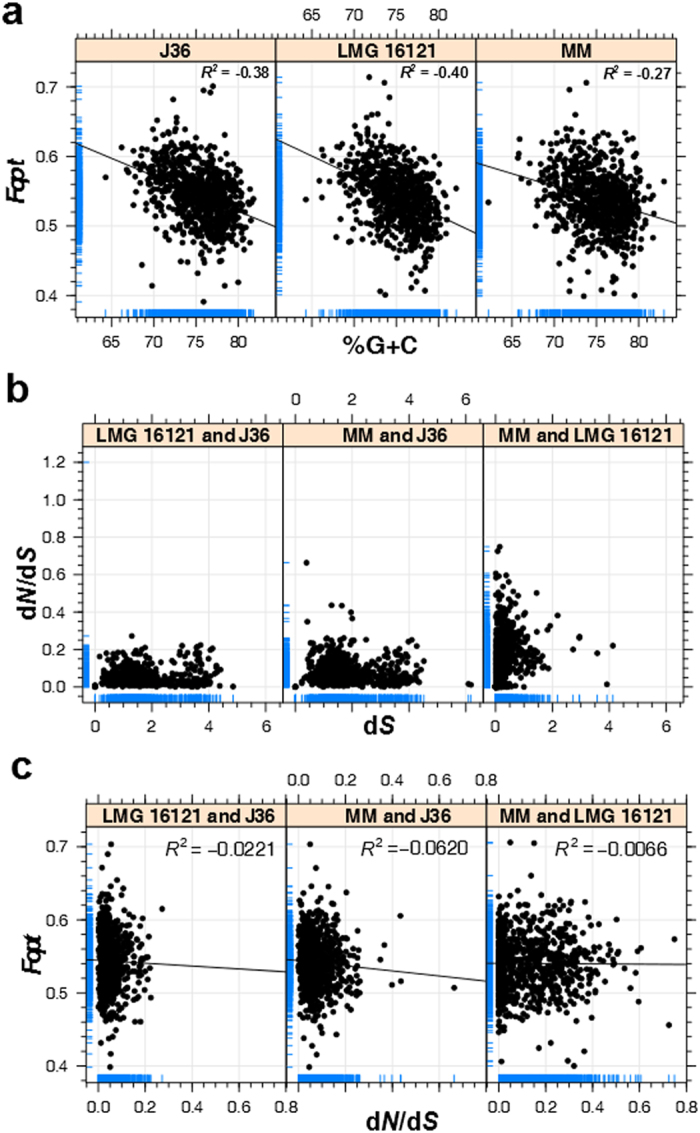
Scatter plot analysis showing coupling between G+C content, *F*_*opt*_ and d*N/*dS across *Cellulosimicrobium* genomes. **(a)** Pearson Product-Moment Correlation between %G+ C and *F*_*opt*_ with labeled R^2^ and *P*-value for *Cellulosimicrobium* sp. strain MM, *Cellulosimicrobium* cellulans LMG16121, and *Cellulosimicrobium* cellulans J36, based on 791 common orthologues between all three genomes. **(b)** d*N*/d*S* values for orthologous proteins in independent pairs of strains *C. cellulans* J36, *C. cellulans* LMG16121, and *Cellulosimicrobium* sp. strain MM. Black dotted line at d*N*/d*S* value of 1 represents the baseline criterion for positive natural selection. **(c)** Pairwise correlation analysis between *F*_*opt*_and d*N*/d*S* values for three genome pairs.

Pairwise correlation between *F*_*opt*_ and %G+ C across the *Cellulosimicrobium* orthologous gene complement (n =  791, see Supplementary Table S4) revealed a weak, but statistically significant (*R*^*2*^ <  − 0.4, *P*-value ≤  3.5e-15) negative correlation (Fig. 2a), which could be interpreted as a result of insufficient codon usage choices due to the 74%G+ C in this genus^17^. The weak-negative correlation between gene-based G+ C content and *F*_*opt*_ can be explained by both high genomic G+ C content (average %G+ C =  74) and G+ C biased gene conversion (gBGC) effect (Fig. 2a)^17^,^18^,^19^. Habitat specific variations were evident (*P*-value <  0.05) in the genome wide pairwise analysis of codon usage across *Cellulosimicrobium* ecotypes. Strain MM (a hot spring ecotype) showed a significantly different codon usage profile when compared with the mesophilic LMG16121 (*P-*value =  5.373e-06) and J36 (*P-*value =  4.722e-07) (see Supplementary Fig. S3) highlighting the differential impact of local environmental functional constraints.

An average d*N/*d*S* of ≤ 0.8 for the 791 orthologous gene pairs revealed that the *C. cellulans* core genome was evolving under purifying selection (Fig. 2b), which is to be expected as these are all essential genes and the genome is G+ C rich^18^. A negative pairwise correlation was observed between *F*_*opt*_ and d*N*/d*S* values for all combinations (Fig. 2c), suggesting an association between the selection of protein sequences and the optimization of codon frequencies.

### Anaerobic respiration leads to *Cellulosimicrobium* population segregation for hot spring ecotypes

**Figure 3:**
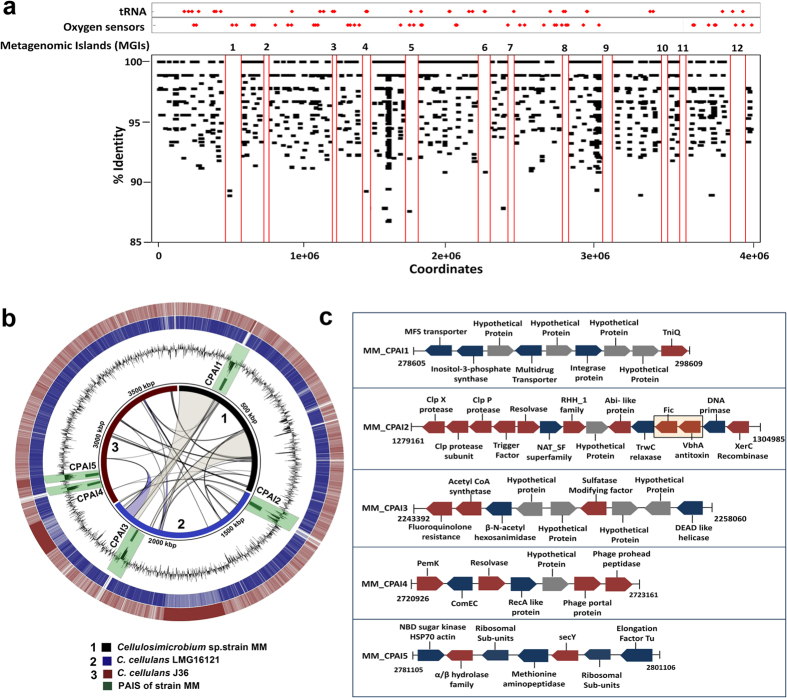
Illustration of pathogenicity islands and metagenomic islands across *Cellulosimicrobium* sp. strain MM. **(a)** Twelve MGIs depicted across the genome of *Cellulosimicrobium* sp. strain MM after mapping of metagenomic reads from biofilm at Manikaran hot springs. **(b)** Whole genome alignments. Rings from inside to outside**: 1**, Whole genome synteny plot of three *Cellulosimicrobium* genomes using 5 kb window size. Black, blue and red rings represent whole genome sequences for strain MM, LMG16121, and J36. BLASTN comparisons of strain MM with two reference genomes i.e. strains LMG16121 and J36: **2**, Black solid represents the genome sequence of strain MM. **3**, Green colored rings represent the location of 5 PAIs deciphered across strain MM. **4**, Circular black line graph shows %G+ C content of strain MM with regions highlighted for sudden variability (*P*-value <  0.05) across the extent of 5 PAIs. **5**, Blue ring represents genome sequence of strain LMG16121. **6**, Red ring represents genome sequence of strain J36. **(c)** The schematic representation for the annotation of 5 PAIs deciphered in the genome of *Cellulosimicrobium* sp. strain MM. The direction of the ORFs shows the gene orientation. A standard nomenclature was followed for each PAI belonging to strain MM as MM_CPAI1, MM_CPAI2, MM_CPAI3, MM_CPAI4, MM_CPAI5 where “MM” stands for the strain and “C” stands for the genus *Cellulosimicrobium*. Blue and red colored blocks represent non-virulent and virulent ORFs, respectively as predicted by VirulentPred. Grey colored blocks represent hypothetical proteins. On MM_CPAI2, ORFs for Fic and VbhA following ‘*selfish operon*’ theory are highlighted.

Metagenomic reads from microbial mat at Manikaran hot spring were recruited on the genome of strain MM, whereby the regions of the MM genome that were underrepresented in the metagenome (Metagenomic Islands; MGIs) highlighted the accessory genome and environment-specific genetic repertoire. MGIs maintained 33 ORFs encoding for multiple tRNA synthetases, such as lysyl, aspartyl, isoleucyl, cysteinyl, etc. (Fig. 3a, see Supplementary Tabe S5). tRNAs were also annotated within MGIs, which supports their horizontal gene transfer potential^20^. Besides the greater abundance of ABC transporters and DNA associated proteins, genes encoding quorum sensing, including oxygen sensor proteins, were enriched in the MGIs (see Supplementary Table S6 Fig. 3a). The oxygen sensing machinery included proteins such as FAD linked oxidoreductase (n =  16), histidine kinase (n =  18), NADH-ubiquinone and quinone oxidoreductase (n =  12), ubiquinone and menaquinone biosynthesis (n =  4), and luciferase family oxidoreductase (n =  4) (see Supplementary Table S6). Additionally, pyridine nucleotide-disulfide oxidoreductase dimerization protein (n =  4), LuxR (n =  13), arsenic resistance protein transcriptional regulator (ArsR) (n =  10), glycerol dehydrogenase like oxidoreductase (n =  6), succinate dehydrogenase (n =  11), and fumarate hydratase (n =  5) were also annotated on the MGIs (see Supplementary Table S6). The Manikaran hot spring microbial mats are characterized by both oxic and anoxic micro-niches^21^, hence *Cellulosimicrobium* hot spring ecotypes may use oxygen sensing for niche adaptation. Strain MM maintained the genetic potential for arsenic mediated respiration (Ars operon, 27.11% identity) and detoxification (Arr operon, 27.95%) with respect to *E. coli* K12. The Ars operon (arsA, arsB, arsC, arsD, arsR) can support respiration across oxic and anoxic conditions, whereas, Arr is only aerobic. Hence, we conclude that respiration might be a splitting factor for the *Cellulosimicrobium* hot spring ecotypes, given that these microbial mats possess oxic and anoxic micro-niches and occurrence of an(aerobic) respiration related genes on the accessory genome of strain MM^21^.

### Identification and characterization of PAIs across *Cellulosimicrobium cellulans* ecotypes

Putative PAIs were identified across the 3 genomes by analyzing variations in %G+ C, codon usage patterns (Fig. 3b), and ‘true’ PAIs were assigned using gene content annotation, e.g. tRNA and virulence genes (Fig. 3b,c, Table 1). VirulentPred^22^ supplemented with MP3^23^ predicted 80 virulent ORFs for the PAIs (MM =  19; LMG16121 =  36; J36 =  25; Fig. 3, see Supplementary Figs S4 and S5). Among these virulent proteins, 61% (49/80) are well known to cause human infections and have been associated with other human pathogens such as *Mycobacterium tuberculosis*^24^, *Staphylococcus aureus*^25^ and *Pseudomonas aeruginosa*^26^ (Table S1).

The whole genome virulence profile showed that 32% (942/3082) of the total protein sequences of *Cellulosimicrobium* sp. strain MM were pathogenic with a threshold score above 0.2 (using MP3). Similarly, LMG16121 had 31% (998/3217) and J36 had 28% (784/2770) pathogenic proteins. It has already been reported that pathogens including, *Mycobacterium tuberculosis* H37Rv, *Pseudomonas aeruginosa* B136–33, *Vibrio cholera* IEC224, and *Neisseria menigitides* 053422, maintain 30.28, 26.2, 18, and 16.3%, respectively. This suggests that *Cellulosimicrobium* carries a high pathogenic potential. A total of 25–28% of the *Cellulosimicrobium* pathogenic genes could be annotated using the KEGG GENES database (see Supplementary Table S7), with 155 genes shared across all three genomes (see Supplementary Table S8). These core pathogenic genes included UDP-N-acetylmuramate dehydrogenase (murB), chitinase, penicillin amidase, ABC transporters (n =  41), multidrug resistance proteins (emrB, n =  4), and drug exporter proteins (n =  3) (see Supplementary Table S8). These common pathogenic genes were also assigned COG classes, whereby 20% were unknown function, 18% were involved in carbohydrate metabolism and transport, 9% in defense mechanisms, 5% in cell wall/membrane/envelope biogenesis, and 2.5% in cell motility (see Supplementary Fig. S6). Cell motility especially was variably distributed across the 3 strains, being nearly absent in J36 (see Supplementary Fig. S6). Interestingly, all the above pathways have recently been proposed as drug targets against *Brucella melitensis* 16M^27^, and therefore may present possible drug targets for treating *Cellulosimicrobium* infections.

The PAI gene content specific to each strain is outlined in the Supplemental information (see Supplementary Text S1) and Fig. 1 (also see Supplementary Figs S4 and S5). Strain MM had some interesting examples, including locus MM_CPAI1 which maintained mobile genetic elements along side multidrug efflux proteins and inositol synthesis, and MM_CPAI2 which maintained genes encoding for anti-toxin VbhA and Fic (filamentation induced by cyclic AMP) proteins (Fig. 3c). Fic are effector proteins which work in a complex with VbhT (toxin) and VbhA (anti-toxin) system^28^. MM_CPAI2 was marked by the presence of juxtaposing Fic and VbhA proteins, speculating that conjugative systems are transferred together via HGT on the pathogenicity or genomic islands loci following the ‘selfish operon model’ (SOM) (Fig. 3c)^8^. As an alternative to SOM, occurrence of these functionally coupled ORFs can also be justified by the co-regulation model whereby genes are clustered in an operon by mere rearrangements followed by selection for co-regulation^8^. The presence of fluoroquinolone resistance, and DEAD-box helicase, pemK, comEC coding regions supports the annotation of these regions as essential for pathogenicity. Similarly, LMG16121 PAIs included genes encoding for UDP-glucose pyrophosphorylase, phage-associated proteins, laminarinase, rhamnolipids and β -glucosidase-related glycosidases (see Supplementary Fig. S4). Finally, J36 PAIs encoded for an abundance of mobile genetic elements, hemin ABC transporter protein, tetracyclin/bleomycin/doxorubicin/methyl viologen resistance proteins, and dihydrofolate reductase (see Supplementary Fig. S5).

### Molecular clock analysis of pathogenicity island proteins

**Figure 4:**
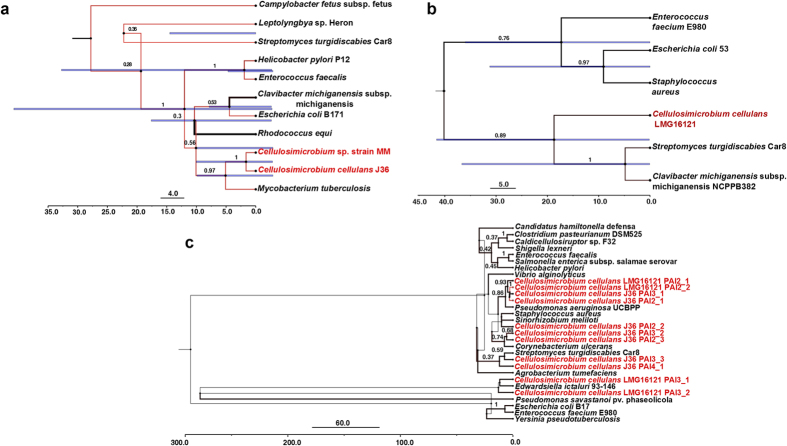
Maximum clade credibility tree summarizing the results of the Bayesian molecular clock analysis of (**a)** Fic protein, (**b)** D-alanyl-D-alanine carboxypeptidase, and (**c)** transposase. The protein sequences of these genes harbored by PAIs of different bacterial lineages were aligned by CLUSTALW and evolutionary rate estimation was performed using BEAST. The timeline indicates the age (mya, million years ago) of nodes. Values above the branches indicate posterior probability values and blue horizontal node bars show the length of the 95% highest posterior density (HPD) interval of node ages. The *Cellulosimicrobium* strains are labeled in red. The branch color gradient (red to black) and width is set according to the increasing substitution rate (r at 95% HPD interval) with black and increased thickness representing the higher substitution rate.

A Bayesian approach was used to calculate the evolutionary protein clock for three PAI proteins, namely Fic, D-alanyl-D-alanine carboxypeptidase and transposase (for selection criterion please see “Methods”)^29^. Molecular clock analysis for the Fic protein (d*N*/d*S* =  1.128) across the multiple bacterial lineages encoding this protein on PAIs (including *Cellulosimicrobium*), predicted the most recent common ancestor (MRCA) to have occurred 27 million years ago (mya) (Fig. 4a). The maximum clade credibility tree revealed *Clavibacter michiganensis* subsp. michiganensis NCPPB382 (r =  0.009 to 4.874) and *Rhodococcus equi* (r =  0.011 to 5.533) with highest substitution rates (r at 95% Highest Posterior Density (HPD) interval) in comparison to rest of the strains (shown by branch thickness and black color, Fig. 4a). While *Clavibacter michiganensis* is well established to be evolving at higher rates using evolutionary dating methodology^30^, *Rhodococcus equi* is also known as an emerging pathogen^31^. The topology of the tree placed *Cellulosimicrobium* strains MM and J36 together and exhibited significant homology (Posterior Probability =  0.97) to that of *Mycobacterium tuberculosis* (Fig. 4a), in which Fic protein has been established to be involved in pathogenicity^32^.

D-alanyl-D-alanine carboxypeptidase found on a PAI in strain LMG16121, has a widespread phylogenetic distribution and a significant role in pathogenesis^33^. The summary tree showed relatively high substitution rates (95% HPD interval) across all the nodes (Fig. 4b). LMG16121 (r =  0.001 to 2.9596) was grouped with *Streptomyces turgidiscabies* (r =  0.004 to 2.8708) and *Clavibacter michiganensis* (r =  0.0001 to 2.6795) (Posterior probability =  0.89). When observed closely (branch thickness), these 3 strains were characterized with relatively lower substitution rates as compared to *Enterococcus faecium* E980 (r =  0.005 to 3.4292), *Escherichia coli* 536 (r =  0.0003 to 3.3744), and *Staphylococcus aureus* (r =  0.005 to 3.4292) (Fig. 4b). The MRCA for D-alanyl-D-alanine carboxypeptidase was calculated at ~40 mya. A similar analysis was also performed across transposase protein sequences from multiple bacterial lineages, including all *Cellulosimicrobium* strains for its frequent presence on PAIs (Fig. 4c). Consistent with previous reports of molecular clock studies of transposable elements^34^, the MRCA for transposase (d*N*/d*S* = 1.06) across the *Cellulosimicrobium* dataset was calculated at ~250 mya, indicating a relative age >100 mya. As expected the majority (n =  11) of the *Cellulosimicrobium* transposases were grouped together, highlighting the species specific nature of this gene family (Fig. 4).

### HGT drives the evolution of PAIs via hypothetical proteins from avirulent isolates

The hypothetical proteins occurring on the PAIs were annotated using ACLAME^35^ which were functionally assigned broadly to mobilization proteins, phage-related proteins, recombinase, and DNA-related proteins (Table 2). All the annotated phage proteins were mapped to prophages or viral peptides in hosts such as *Rhodobacter sphaeroides*, *Paracoccus denitrificans* PD1222, and *Burkholderia pseudomallei*. However, one specific “phage lambda-related host specificity protein J” found on J36_PAI2 was found with origin in plasmid pMT1 of *Yersinia pestis* biovar Microtus str. 91001 (Table 2). Strikingly, only 37% (17/46) of the hypothetical proteins annotated across the 13 PAIs in the *Cellulosimicrobium* pan-genome were predicted to have originated from pathogenic bacteria (Table 2). The remaining 63% were predicted to have originated from non-pathogenic bacterial hosts, which highlights that HGT interactions between pathogens and other bacteria can play a significiant role in the evolution of PAIs. High number of hypothetical proteins has also been found on PAIs of other pathogenic bacteria such as *Pseudomonas aeruginosa* PAO1^16^. PAIs themselves are mobile genetic elements and are well known to have been acquired during speciation of pathogens from non-pathogenic or environmental ancestors. Hence, PAIs harbor both virulent and avirulent ORFs and thus origin of hypothetical proteins can be mapped to avirulent bacterial isolates as well.

### Metagenomic recruitment of PAIs across Manikaran hot springs

**Figure 5:**
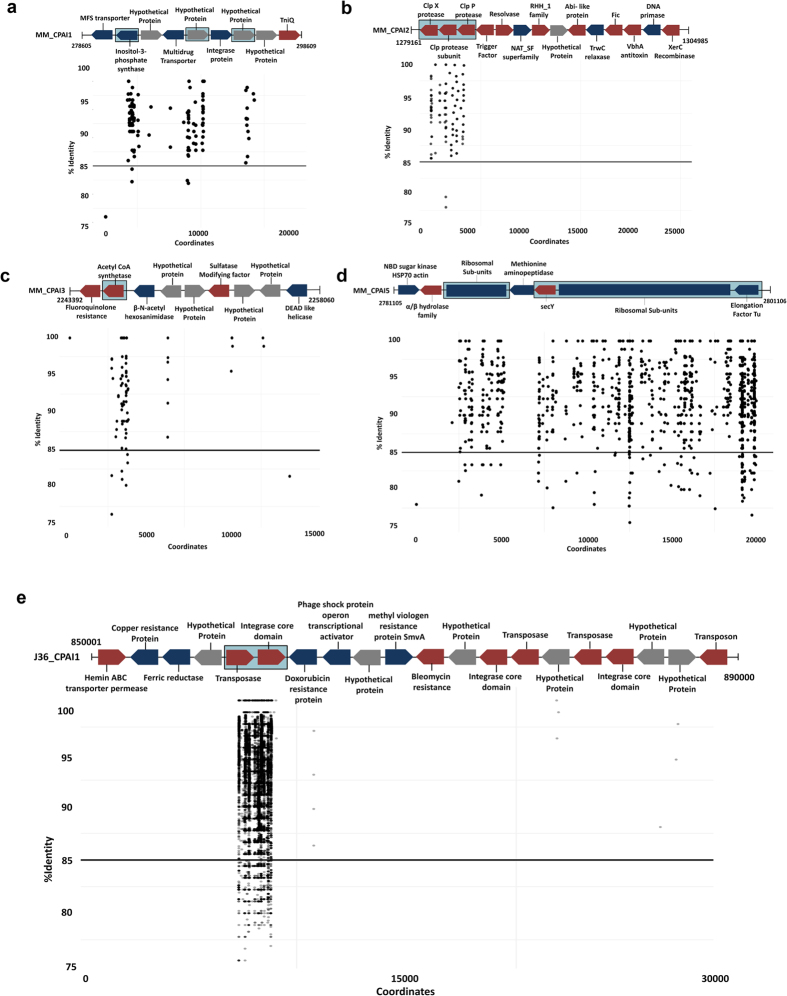
Recruitment plot showing binning of metagenomic reads from biofilm at Manikaran hot springs on pathogenicity islands (PAIs). (**a**) One dot represents each read aligned onto the PAIs of *Cellulosimicrobium* sp. strain MM namely MM_CPAI1, (**b**) MM_CPAI2, (**c**) MM_CPAI3, (**d**) MM_CPAI5, and (**e**) J36_CPAI2 from *Cellulosimicrobium cellulans* J36. *x*-*y* axes represent the sequence co-ordinates and sequence identity, respectively. Blue, red and gray blocks represent non-virulent, virulent (as predicted by VirulentPred) and hypothetical proteins, respectively.

We mapped environmental metagenomic reads from Manikaran hot-spring microbial mats to the 13 PAIs. Only 38% of the PAIs showed significant recruitment, with the majority in strain MM, which was isolated from these microbial mats (Fig. 5). Metagenomic reads mapped to the PAIs were enriched for ORFs encoding for integrase, transposase, secY, inositol-3-phosphate synthase, and Clp subunits (Fig. 5). Inositol-3-phosphate synthase is known to be laterally transferred from Archaea to thermophillic bacteria^36^, and also plays a role in *Mycobacterium tuberculosis* pathogenicity^37^. The mapping of metagenomic reads to the secY protein (Fig. 5), suggests that the hot spring community is experiencing an elevated stress response (with respect to both temperature i.e. 57 °C and arsenic) across this environment^21^. Clp subunits (ClpX and ClpP), which are virulence markers, were also enriched in the microbial mats (Fig. 5)^38^. This suggest that the microbial community is under considerable environmental stress and maintains significant virulence potential.

## Conclusions

*C. cellulans* has been associated with human pathogenicity, which is likely acquired and conferred through mobile genetic elements including pathogenicity islands. Using 3 reference genomes, *C. cellulans* LMG16121, *C. cellulans* J36, and *Cellulosimicrobium* sp. strain MM, we annotated 13 PAIs, encoding 49 potential virulence factors well-established to cause human infections. However, 32% (63/200) of the annotated PAI ORFs encoded for unknown proteins, of which 63% mapped to non-pathogenic bacteria, supporting the role of HGT in the evolution of PAIs^16^. Characterized with a high G+ C content (average 74%), genus *Cellulosimicrobium* was predicted to experience high selection pressure, with an average dN/dS value of 0.8 (< 1) based on 791 orthologous genes. This study provides first insights into the evolution of PAIs across the genus *Cellulosimicrobium* and reveals 49 virulence genes such as, Fic, VbhA toxin/antitoxin system, FtsK, ClpX, etc. which can be used as diagnostic markers for pathogenic *Cellulosimicrobium* strains.

## Methods

### Phylogenomic analysis

Phylogenomic analysis was performed in order to assign phylogenetic status to the uncharacterized (at species level) *Cellulosimicrobium* strain MM. Given a limited number of genomes sequenced for the genus *Cellulosimicrobium* (n =  4), we used dataset from the complete family *Promicromonosporacae* (n =  8). 16S rRNA gene sequence, 31 single copy genes^10^ and 400 conserved marker protein sequence based methods^11^ were used to perform the phylogenetic analysis for the family. The 16S rRNA gene sequences (n =  31) were retrieved from the NCBI database for all seven genera included in family *Promicromonosporacae* namely: *Cellulosimicrobium*, *Isoptericola*, *Myceligenerans*, *Promicromonospora*, *Xylanibacterium*, *Xylanimicrobium*, and *Xylanimonas* including *Cellulomonas aerilata* 5420S-23 as the outgroup. Similarly, phylogenomic reconstruction was performed based on amino acid sequences of 400 conserved bacterial markers and 31 single copy genes belonging to eight genome sequences of the family *Promicromonosporacae* from NCBI database viz. *Cellulosimicrobium cellulans* J36, *Cellulosimicrobium cellulans* LMG16121, *Cellulosimicrobium* sp. strain MM, *Cellulosimicrobium funkei* U11, *Isoptericola variabilis* strain 225, *Promicromonospora kroppenstedtii* DSM 19349, and *Promicromonospora sukumoe* 327MFSha31, and *Xylanimonas cellulosilytica* DSM 15894 (http://www.ncbi.nlm.nih.gov/genome/browse/representative/). The concatenated sequences were aligned using CLUSTALW^39^ and a maximum likelihood^40^ tree was constructed at bootstrap value of 1000, implemented in software MEGA 6.0^41^. Further, in order to support 16S rRNA and marker gene based phylogenetic analysis, ANI and DDH values^9^ were also calculated for the 8 whole genomes from *Promicromonosporacae.* ANI was calculated at minimum alignment length cut-off of 700 bp, minimum identity cut-off of 70% and window size of 1000 bp.

### Functional annotations and metagenomic recruitment of *Cellulosimicrobium* strains

To determine the functional variability across three *Cellulosimicrobium* strains, i.e. MM, LMG16121 and J36, comparative genomic analysis was performed based on proteins and metabolic pathways. ORFs were predicted from the three genome sequences using FragGeneScan 1.18^42^ followed by gene finding using KAAS (KEGG Automatic Annotation Server) by BLASTp against the KEGG (Kyoto Encyclopedia of Genes and Genomes) GENES database at E-value of 1e-5 and identity cut-off of 70%. Metabolic pathways were reconstructed and filtered using MinPath (Minimal set of Pathways)^43^ for all the *Cellulosimicrobium* genomes, i.e. strains MM, LMG16121, and J36. Further, one-way hirerachical clustering was performed on the top 50 variables (i.e. metabolic pathways) and were plotted at relative abundance of 0.8% and standard deviation cut-off of 0.4%. Protein orthologues were determined using pairwise reciprocal smallest distance (RSD) algorithm initially, followed by retrieving the common set in all three genomes^44^ at E-value of 1e-15 and divergence cut-off of 0.5. Whole genome alignments were created in Mauve 2.4.0^45^ and visualized in Circos^46^ at 10 Kb minimum cut-off. In order to elucidate the effect of arsenic contamination across microbial mats, arsenic related gene clusters were reconstructed from strain MM’s genome using PSI BLAST and identity cut-off of 25%.

HGT events were determined for three *Cellulosimicrobium* genomes based on codon usage deviation using Hidden Markov models (HMM) implemented in program SIGI-HMM^47^. Hierarchical clustering was performed on the annotated HGT loci using Euclidean distance matrix across three *Cellulosimicrobium* genomes. Putative alien (pA) genes were hence determined on the stretch of genomic islands (GIs) using Viterbi algorithm based on codon usage variations of HGTs from the rest of the genome^47^. Paired-end metagenomic reads (n =  78,891,278) from the biofilms at Manikaran hot springs^21^ were mapped over the genome of strain MM using GASSST (Global Alignment Short Sequence Search Tool)^48^ at sequence similarity cut-off of 85% to allow for *Cellulosimicrobium* strain MM specific recruitments^49^. Abundance-weighted average coverage analysis using Nonpareil^50^ revealed that the metagenome dataset had a genome coverage of 93% against strain MM. Therefore, we believe that our sequencing depth was good enough to represent the *in situ* microbial diversity.MGIs were annotated as continuous stretches of gaps in the metagenome recruitment plot by subjecting them to ORF prediction and further BLASTp against the NCBI nr database at E-value of 1e-5, minimum bit-score cut-off of 100 and identity cut-off of 70%^49^.

### Determination of pathogenicity islands (PAIs)

Three genomes representing *C. cellulans* i.e. strains LMG16121, MM and J36 were used for deciphering PAIs following a segregative approach discriminating islands from the core genome on the basis of %G+ C content, dinucleotide frequency, and codon usage implemented in program PAI-DA^20^,^51^ with window size of 5 Kb per genome. Both %codon usage bias and %G+ C content were plotted against the individual genome to identify skewed “island-like” regions. Simultaneously, PAI-DB v2.0^52^ was used to validate occurrence of these PAI like regions against databases for both pathogenicity islands (PAIs) and resistance islands (REIs) at E-value of 0.01. Given that highly expressed genes (HEGs) such as genes encoding for ribosomal subunits, transcription and terminator genes and repair genes might also exhibit a skewed codon usage and G+ C content^20^, we used sequence composition information (manual curation) to avoid the false negative predictions. Further, tRNA scan was used to check whether PAIs are flanked by tRNAs to validate the PAIs^53^.

### Assigning functions to PAIs

Protein coding sequences were obtained for the PAIs of all three genomes by FragGeneScan 1.18^42^. Functions were assigned to the ORFs using BLASTp (E-value =  1e-5) against non-redundant protein database, KEGG^54^ and Gene Ontology (GO) (Gene Ontology Consortium, 2008) database. Protein sequences were also searched against the Pfam library of hidden Markov models (HMMs) using HMMER^55^ for family level prediction. Further, the virulent content of the PAIs was determined using multiple databases such as Virulent Factor Database (VFDB)^56^, VirulentPred^22^, and MP3^23^. MP3 was used at a threshold value of 0.2 and minimum protein length of 30.The hypothetical proteins which were abundant on PAIs were then checked for their origin using the ACLAME database^35^.

### Molecular clock analysis of PAI gene content

After functional assignment of ORFs predicted on PAIs of the three genomes, we selected three proteins, i.e. Fic, D-alanyl-D-alanine carboxypeptidase, and transposase from strain MM, LMG16121 and J36, respectively, to infer the divergence time of most recent common ancestor (MRCA) among multiple bacterial lineages harboring these genes on PAIs. In case where multiple strains of one bacterial species were found to be carrying the gene of interest on PAIs, only single strain was taken into account for tree construction. Fic protein from strain MM was selected for its significant association with pathogenesis^57^ as well as its frequent occurrence on PAIs in other pathogenic bacterial lineages (n =  9). PAIs from strain LMG16121 were characterized by the repeated presence (n =  9) of ORFs encoding for proteins involved in cell-wall biogenesis out of which D-alanyl-D-alanine carboxypeptidase was chosen for its association with PAIs of other pathogenic bacteria (n =  5). Transposase was also selected for this analysis, for its repeated presence on PAIs of strain J36 (n =  7) as well as forming a significant part of PAIs in other bacterial genera (n =  19). The protein coding sequences for each of these genes (n =  3) was retrieved from *Cellulosimicrobium* genomes (n =  3) as well as from other bacteria (NCBI) reported for the presence of these genes on PAIs followed by multiple sequence alignment using CLUSTALW. The alignment was then used in BEAST version 1.8.2^29^ to perform Bayesian molecular clock analysis using the following parameters: Clock =  Random Local Clock, Substitution model =  WAG (for amino acids), Site heterogeneity =  Gamma, Tree Prior =  Coalescent Constant Size, Length of Monte Carlo Markov Chain (MCMC) =  1000000 and Burnin =  100. Random Local Clock and Gamma distribution (relaxed model) was used to account for maximum heterogeneity in terms of substitutions given inter-genera, diverse nature of the dataset. TreeAnnotator (http://beast.bio.ed.ac.uk/TreeAnnotator) was further used to summarize the information from multiple sample trees generated from BEAST into a single target tree, i.e. “Maximum clade credibility” tree with values for the rate of substitution at 95% Highest Posterior Density (HPD) interval, posterior probability, length and height of 95% HPD interval of the node ages. The final annotated tree was then visualized using FigTree version 1.4.0 (http://tree.bio.ed.ac.uk/software/figtree/).

### Statistical analysis

For *Cellulosimicrobium* genomes undertaken in this study, pairwise correlation was computed between gene centric optimal codon frequencies *F*_*opt*_ (a measure of codon usage bias) and %G+ C content using Pearson Product-Moment Correlation Coefficient (*R*^*2*^) at 95% confidence level based on 791 common orthologues found between three genomes^17^. *F*_*opt*_ values were calculated using CodonW (version 1.4.4, http://codonw.sourceforge.net) for the 791 orthologous proteins from each of three *Cellulosimicrobium* genomes. Further, pairwise comparisons between the codon usage bias (measured as *F*_*opt*_ values) were performed between *Cellulosimicrobium* sp. MM, *C. cellulans* LMG16121 and *C. cellulans* J36, using Wilcoxon-Mann-Whitney test with continuity correction to elucidate the significance of variable codon bias patterns across different genomes inhabiting different environments^58^. To further estimate coupling between selection on codon usage and selection of amino acids, Pearson correlation was computed between *F*_*opt*_ and the d*N*/d*S* for orthologous gene pairs (n =  791) between all three genome pairs. For this the mean *F*_*opt*_ value of two orthologous genes was taken as the *F*_*opt*_ value for that gene pair. d*N*/d*S* values for each orthologous gene pair was calculated by pairwise aligning protein sequences by CLUSTALW followed by codon to codon alignment of corresponding nucleotide sequences using PAL2NAL^59^. Further substitution rates were estimated using yn00 module implemented in PAML^60^. All the above statistical analyses and scatter plotting were performed in R (R Core Team, http://www.R-project.org/).

### Accession numbers

Sequence data were obtained for the *Cellulosimicrobium* genomes from NCBI Genome database: *Cellulosimicrobium* sp. strain MM [GenBank:NZ_JPQW00000000.1], *Cellulosimicrobium cellulans* LMG16121 [NZ_CAOI00000000.1], *Cellulosimicrobium cellulans* J36 [NZ_JAGJ00000000.1], *Cellulosimicrobium funkei* U11 [NZ_JNBQ00000000.1], *Isoptericola variabilis* 225 [NC_015588.1], *Promicromonospora kroppenstedtii* DSM 19349 [NZ_AZXR00000000.1], *Promicromonospora sukumoe* 327MFSha31 [NZ_ARQM00000000.1], and *Xylanimonas cellulosilytica* DSM 15894 [NC_013530.1]. 16S rRNA gene sequence data was used from the family *Promicromonosporacae* under accession numbers: *Promicromonospora citrea* [X83808.1], *Promicromonospora endophytica* EUM 273 [GU434253.2], *Promicromonospora enterophila* [X83807.1], *Promicromonospora flava* [AM992980.1], *Promicromonospora* sp. UTMC 792 [JN038073.1], *Promicromonospora sukumoe* [AB023375.1], *Promicromonospora thailandica* [AB560974.1], *Promicromonospora kroppenstedtii* RS16 [AM709608.1], *Promicromonospora aerolata* [AJ487303.1], *Promicromonospora umidemergens* [FN293378.1], *Promicromonospora vindobonesis* [AJ487302.1], *Promicromonospora xylanilytic*a strain YIM61515 [FJ214352.1], *Cellulosimicrobium cellulans* [X83809.1], *Cellulosimicrobium funkei* strain W6122 [AY501364.1], *Cellulosimicrobium terreum* strain DS-61 [EF076760.1], *Isoptericola chiayiensis* strain 06182M-1 [FJ469988.1], *Isoptericola dokdonensis* strain DS-3 [DQ387860.1], *Isoptericola halotolerans* strain YIM 70177 [AY789835.1], *Isoptericola hypogeus* [AJ854061.1], *Isoptericola jiangsuensis* strain CLG [EU852101.1], *Isoptericola nanjingensis* strain H17 [HQ222356.1], *Myceligenerans crystallogenes* [FR733716.1], *Myceligenerans* sp. XJEEM 11063 [EU910872.1], *Myceligeneris xiligouensis* strain XLG9A10.2 [AY354285.1], *Xylanibacterium ulmi* [AY273185.2], *Xylanimicrobium pachnodae* VPCX2 [AF105422.1], *Xylanimonas cellulosilytica* DSM 15894 [CP001821.1], and *Cellulomonas aerilata* strain 5420S-23 [EU560979.1]. Metagenome sequence (NGS) data were obtained from DDBJ/EMBL/GenBank under the accession number of PRJEB4614 (http://www.ebi.ac.uk/ena/data/view/PRJEB4614).

## Additional Information

**How to cite this article**: Sharma, A. *et al.* (Meta)genomic insights into the pathogenome of *Cellulosimicrobium cellulans. Sci. Rep.* **6**, 25527; doi: 10.1038/srep25527 (2016).

## Supplementary Material

Supplementary Information

Supplementary Dataset S4

Supplementary Dataset S5

Supplementary Dataset S6

Supplementary Dataset S7

Supplementary Dataset S8

## Figures and Tables

**Table 1:** General features of PAIs determined across three *Cellulosimicrobium* genomes.

S.No.	PAI designation	Start	End	Size (in Kbp)	Codon Usage Bias	%G+ C Difference	Number of ORFs predicted	Annotated Proteins	Hypothetical proteins	Metagenome recruitment
*Cellulosimicrobium* sp. strain MM										
1.	MM_PAI1	278605	298609	20	0.17	0.063	15	9	4	Yes
2.	MM_PAI2	1279161	1304985	25.8	0.194	0.038	29	14	1	Yes
3.	MM_PAI3	2243392	2258060	14.7	0.21	0.057	11	9	4	Yes
4.	MM_PAI4	2720926	2723161	2.2	0.168	0.054	12	7	1	No
5.	MM_PAI5	2781105	2801106	20	0.124	0.048	28	7	0	Yes
*Cellulosimicrobium cellulans* LMG16121										
1.	LMG_PAI1	2985001	3010000	25	0.281	0.072	14	13	0	No
2.	LMG_PAI2	3460001	3480000	20	0.198	0.039	18	14	7	No
3.	LMG_PAI3	3490001	3495000	5	0.203	0.035	6	6	4	No
4.	LMG_PAI4	3500001	3550000	50	0.216	0.046	48	42	15	No
5.	LMG_PAI5	4220001	4230000	10	0.23	0.028	8	8	3	No
*Cellulosimicrobium cellulans* J36										
1.	J36_PAI1	850001	890000	40	0.285	0.068	26	20	6	Yes
2.	J36_PAI2	915001	970000	55	0.233	0.037	57	41	18	No
3.	J36_PAI3	1445001	1460000	15	0.221	0.05	10	10	0	No

**Table 2:** Annotation of hypothetical proteins deciphered on PAIs across three *Cellulosimicrobium* genomes using ACLAME database.

S.No.	ACLAME Annotation	Origin	Host	MGE class	Coordinates
*Cellulosimicrobium* sp. strain MM					
CPAI2					
1.	Putative MrcB penicillin binding protein B	Plasmid; pSymA	*Sinorhizobium meliloti* 1021	607	3768, 6348
2.	Hypothetical protein	Plasmid; pRHL1	*Rhodococcus* sp. RHA1	814	16606, 17598
CPAI3					
3.	Putative outer membrane protein	Plasmid; pKPN3	*Klebsiella pneumoniae* subsp. *pneumoniae* MGH 78578	1959	7084, 8875
4.	Mobilization protein	Plasmid; pKJ50	*Bifidobacterium longum*	247	8912, 9427
5.	Orf15	Viral peptides	*Haemophilus influenza*	328	11205, 11626
6.	Hypothetical protein	Plasmid	*Anabaena variabilis* ATCC 29413	1145	12071, 13867
CPAI4					
7.	Phage terminase	Prophage	*Rhodobacter sphaeroides* ATCC 17029	2612	9596, 10915
*Cellulosimicrobium cellulans* LMG16121					
CPAI3					
8.	Hypothetical protein	Plasmid; pREL1	*Rhodococcus erythropolis* PR4	773	1993, 2385
9.	Putative atp/gtp-binding protein	Plasmid	*Streptomyces coelicolor*	656	5790, 7312
10.	Hypothetical protein	Plasmid	*Arthrobacter aurescens* TC1	1812	9579, 10760
11.	Type I site-specific deoxyribonuclease, HsdR family	Plasmid; pPNAP05	*Polaromonas naphthalenivorans* CJ2	1820	10905, 11417
12.	Hypothetical protein	Plasmid; ColIb-P9	*Shigella sonnei*	515	11857, 12980
13.	Hypothetical protein	Plasmid; pBD2	*Rhodococcus erythropolis*	579	17843, 19254
CPAI4					
14.	Hypothetical protein	Plasmid	*Streptomyces coelicolor*	656	247, 1968
15.	Conjugal transfer protein	Plasmid; pXF51	*Xylella fastidiosa* 9a5c	686	2269, 3240
CPAI5					
16.	Hypothetical protein	Plasmid; pREL1	*Rhodococcus erythropolis*	773	5006, 5892
17.	Site-specific recombinase for integration and excision	Viral peptides; phi-105	*Bacillus subtilis*	329	7307, 7675
18.	Hypothetical protein	Plasmid; pREC1	*Rhodococcus erythropolis* PR4	1126	8234, 8950
19.	Putative transcriptional regulator	Plasmid; pCM2	*Clavibacter michiganensis* subsp. *michiganensis* NCPPB 382	1957	8984, 10622
20.	Integrase	Prophage	*Burkholderia pseudomallei* 668	2619	11024, 11988
21.	Hypothetical protein	plasmid; pMFLV02	*Mycobacterium gilvum* PYR-GCK	1958	12080, 12985
22.	Hypothetical protein	plasmid; pREL1	*Rhodococcus erythropolis* PR4	773	13074, 14654
23.	Hypothetical protein	Plasmid	*Arthrobacter* sp. FB24	786	16493, 16714
24.	Hypothetical protein	plasmid; pRL11	*Rhizobium leguminosarum bv. viciae* 3841	741	35361, 35924
25.	Hypothetical protein	plasmid; pRL12	*Rhizobium leguminosarum* bv. viciae 3841	779	35967, 36521
26.	Hypothetical protein	plasmid; pSymA	Sinorhizobium *meliloti* 1021	607	41413, 41835
CPAI6					
27.	Transfer gene complex protein-like protein	plasmid; p103	*Rhodococcus equi*	576	408, 1903
28.	Integral membrane protein, putatine	plasmid; TC2	*Arthrobacter aurescens* TC1	1812	1916, 2799
29.	Putative septum site-determining protein (MinD)	plasmid; pBD2	*Rhodococcus erythropolis*	579	5987, 7340
*Cellulosimicrobium cellulans* J36					
CPAI1					
30.	Hypothetical protein	Plasmid	*Arthrobacter* sp. FB24	715	11725, 11943
31.	Clp N terminal domain protein	Plasmid; pMFLV02	*Mycobacterium gilvum* PYR-GCK	1958	19589, 19837
32.	Type IV secretion/conjugal transfer ATPase	Plasmid	*Burkholderia cepacia* AMMD	910	27425, 31462
33.	Hypothetical protein	Plasmid	*Nitrobacter hamburgensis* X14	714	35373, 35855
CPAI2					
34.	Phage tail tape measure protein	Viral peptides; phiN315	*Staphylococcus aureus subsp. aureus* N315	170	6168, 6698
35.	Putative alkylmercury lyase	Plasmid	*Arthrobacter* sp. FB24	752	6714, 7025
36.	Putative DNA primase/helicase	plasmid; pSLA2-L	*Streptomyces rochei*	661	11012, 11650
37.	DNA primase catalytic core	plasmid; pNOCA01	*Nocardioides* sp. JS614	1808	11656, 14345
38.	Hypothetical protein	Plasmid	*Arthrobacter* sp. FB24	786	14933, 15175
39.	TraF/VirB10-like protein	plasmid; pF1947	*Haemophilus influenzae biotype aegyptius*	720	17652, 18719
40.	Transfer gene complex protein-like protein	plasmid; p103	*Rhodococcus equi*	576	25494, 26919
41.	Putative secreted protein	plasmid; pCM2	*Clavibacter michiganensis subsp. michiganensis* NCPPB 382	1957	27007, 27747
42.	Phage lambda-related host specificity protein J	plasmid; pMT1	*Yersinia pestis biovar Microtus* str. 91001	1123	36826, 38202
43.	LtrC-like protein	plasmid; pC4602-2	*Vibrio vulnificus*	1968	43611, 44552
44.	Hypothetical protein	plasmid; pCC7120epsilon	*Nostoc* sp. PCC 7120	495	44756, 45819
45.	Phage-related protein	viral peptides	*Clostridium botulinum* C	1466	52588, 53604
