# Supplementary material for: (Meta)genomic insights into the pathogenome of Cellulosimicrobium cellulans
Source: Sci Rep. 2016 May 6;6:25527. doi: 10.1038/srep25527 (PMC4858710; doi:10.1038/srep25527)
Supplement: Supplementary Information [file srep25527-s1.pdf]

(Meta)genomic insights into the pathogenome of *Cellulosimicrobium cellulans*.

Anukriti Sharma[a], Jack A. Gilbert[b,c,d], and Rup Lal[a*]

Department of Zoology, University of Delhi, Delhi, India[a].

Biosciences Division (BIO), Argonne National Laboratory, 9700 South Cass Avenue, Argonne, IL, U.S.A[b].

Department of Surgery, University of Chicago, 5841 S Maryland Ave, Chicago, IL, U.S.A[c].

Marine Biological Laboratory, Woods Hole, MA, U.S.A[d].

Legends of Supplementary Information

Text S1: The PAI gene content specific to each strain is outlined in this section.

Figure S1: Rooted Maximum likelihood tree based on Jukes-Cantor model for family *Promicromonosporacae* using 400 bacterial marker genes with *Cellulosmonas flavigena* DSM 20109 as outgroup. The percentage (>70%) of replicate trees in which the associated taxa clustered together in the bootstrap test (1000 replicates) are shown next to the branches. All the trees are drawn to scale, with branch lengths measured in the number of amino acid substitutions per site.

Figure S2: Heatmap with column dendrogram using hierarchical clustering based on HGT gene content using Euclidean distance matrix.

Figure S3: (a) The pairwise Wilcoxon ranked sum (V) and *P*-values across *Cellulosimicrobium* genomes (n = 3) with respect to Codon bias (F_{opt}) distribution using the Wilcoxon-Mann-Whitney test**. (b)** Schematic representation of phage-like region determined from the genome of strain MM.

Figure S4: The schematic representation of ORFs annotated on five PAIs across genome of *Cellulosimicrobium cellulans* LMG16121. The direction of the ORFs shows the gene orientation. Nomenclature followed for each PAI belonging to strain LMG16121 is written as (a) LMG_CPAI1, (b) LMG_CPAI2, (c) LMG_CPAI3, (d) LMG_CPAI4, and (e) LMG_CPAI5 where "LMG" stands for the strain and "C" stands for the genus *Cellulosimicrobium*. Gray and red colored blocks represent non-virulent and virulent ORFs respectively. Slightly shaded colored blocks represent hypothetical proteins.

Figure S5: The schematic representation of ORFs annotated on 3 PAIs across genome of *Cellulosimicrobium cellulans* J36. The direction of the ORFs shows the gene orientation. A standard nomenclature was followed for each PAI belonging to strain J36 as (a) J36_CPAI1, (b) J36_CPAI2, and (c) J36_CPAI3 where "J36" stands for the strain and "C" stands for the genus *Cellulosimicrobium*. Grey and red colored blocks represent non-virulent and virulent ORFs respectively. Slightly shaded colored blocks represent hypothetical proteins.

Figure S6: Distribution of COG classes (in percentage) across the pathogenic gene complements of the three genomes i.e. *Cellulosimicrobium* sp. strain MM, *Cellulosimicrobium cellulans* LMG16121, and *Cellulosimicrobium cellulans* J36.

Table S1 (Doc file): List of proteins annotated across PAIs from all *Cellulosimicrobium* ecotypes, established to be associated with human infections.

Table S2 (Doc file): Pairwise ANI values between 8 genomes of family *Promicromonosporacae*.

Table S3 (Doc file): DNA-DNA hybridization (DDH) values calculated by GGDC for pairwise combinations of *Cellulosimicrobium* sp. strain MM and other 7 genomes.

Table S4 (Excel File): Annotations of 598 out of 792 common orthologues between *Cellulosimicrobium* sp. strain MM, *C. cellulans* LMG16121, *C. cellulans* J36.

Table S5 (Excel File): Relative abundance of HGT events across *Cellulosimicrobium* sp. strain MM, *C. cellulans* LMG16121, *C. cellulans* J36**.**

Table S6 (Excel File): Annotation of 12 MGIs using *Cellulosimicrobium* sp. strain MM as reference genome**.**

Table S7 (Excel File): Annotation of pathogenic proteins predicted for *Cellulosimicrobium* sp. strain MM, *C. cellulans* LMG16121, and *C. cellulans* J36.

Table S8 (Excel File): List of common pathogenic proteins across three genomes i.e. *Cellulosimicrobium* sp. strain MM, *C. cellulans* LMG16121, *C. cellulans* J36.

Supplementary Information

Text S1

The PAI gene content specific to each strain:

(a) PAIs of *Cellulosimicrobium* sp. strain MM

The first PAI (locus; MM_CPAI1) (Figure 2A, 2B, Table 1) was characterized by the presence of genes for hypothetical proteins, integrase, transposase (TniQ), inositol-3-phosphate synthase and multidrug efflux transporter protein. Inositol is required for synthesis of phospholipid in both prokaryotes and eukaryotes [46] especially by pathogens with a desperate need to be able to proliferate inside hosts. Human pathogens are known to acquire inositol either via synthesis or import from host fluids (such as blood). Despite the abundance of inositol in host environments, certain pathogenic bacteria such as Mycobacteria (Actinobacteria) synthesisize inositol via enzymes inositol-3-phosphate synthase [46].

The second locus (MM_CPAI2, Figure 2A, 2B, Table 1) was characterized by Clp proteolytic subunits consisting of an enzyme system responsible for proteolysis of misfolded/damaged proteins. These proteins are well-established markers of *M. tuberculosis* pathogenesis [47]. Besides the presence of gene encoding site recombinase

such as XerC, genes encoding for anti-toxin VbhA and Fic (filamentation induced by cyclic AMP) proteins were also annotated across the second locus (MM_CPAI2) (Figure 2B). Fic proteins are effector proteins which work in a complex with VbhT (toxin) and VbhA (anti-toxin) system, utilized by pathogenic bacteria to interfere with cell signaling of the host cell collapsing the actin cytoskeleton, hence cell death [3]. This PAI hence implicitly demonstrated the 'selfish operon' theory marked by the presence of juxtaposing Fic and VbhA proteins, speculating that conjugative systems are transferred together on the pathogenicity or genomic islands loci. One of the ORFs was predicted to be TrwC Relaxases which work with the cognate conjugative system Type IV secretion system (T4SS) and are bona fide virulence factors [2] (Figure 2B). In addition to the above mentioned virulence factors, ORF for bacteriocin-like protein was also annotated which represents a class of proteins possessing bactericidal activity modulating both inter-strain and inter-species inhibition [48].

Microbial antibiotic resistance is not a pathogenicity factor; however, it helps in enhancing the virulence/fitness across environments with increased chemical stress [49]. The third PAI locus (MM_CPAI3) consisted of genes for fluoroquinolone resistance, which is already known to be a marker trait of extra-intestinal pathogenic bacteria such as *Escherichia coli* [5] (Figure 2B, Table 1). Discounting four hypothetical proteins, gene encoding for sulfatase modifying factor was also found on this locus, which has been reported to be responsible for increased fitness of pathogenic bacteria such as *Streptococcus pneumonia* using mutational analysis [6]. Interestingly, one of the ORFs was annotated (Figure 2B, Table 1) as DEAD-box helicase, which has been documented earlier on one of the O islands in formidable pathogen *E. coli* O157:H7. Using deletion

mutant assay, DEAD-box helicase was reported to impede the function of *fliC* gene responsible for flagellar driven motility [50].

Strikingly, ORFs on the fourth locus (MM_CPAI4, Figure 2B, Table 1) encoded for virulence determinants such as pemK, comEC, phage related genes and resolvase. pemK genes form a part of toxin-antitoxin (TA) system which has been implicated in modulation of virulence in pathogenic bacterial strains such as *Mycobacterium tuberculosis*, *Staphylococcus aureus*, *Yersinia pestis*, *Helicobacter pylori*, *Streptococcus mutans* and *Clostridium difficile*, however the exact mechanism still needs experimental verification [51]. ComEC (competence) genes are primarily responsible for sizeable foreign DNA uptake and are favorably present in pathogenic bacteria in contrast to non-pathogenic bacteria to boost the competence [52]. Moreover, the presence (Figure 2B) of phage remnants implicates the virulence via phage-encoded pathogenicity factors [53]. Gene centric annotation of strain MM revealed a continuous 17.3 Kb long phage like region (ORFs = 18) with best match to phages like Macaci herpesvirus and Pandor dulcis (Figure S1B).

Annotation of the fifth locus (MM_CPAI5) elucidated ORFs for ribosomal subunits, elongation factor TU and *sec*Y (Figure 2B). *secY* specifically forms translocation channel for the transportation of pre-proteins across the bacterial membrane. Translation initiation factor-1 (Translation IF1) and elongation factor TU were also annotated on this island, which can be challenged owing to skewed G+C content and codon usage of HEGs. However, Fernández-Gómez *et al.*, showed that islands besides harboring foreign genes could also acquire basic transcription and regulation factors for modulating bacterial survival under varied environmental conditions [54]. The fact that MM_CPAI5 is a true

island (Figure 2B) is evidenced by the existence of same gene cassette on plasmid found in *Shewanella baltica* OS155 (pSbal03) which was exclusively absent on the chromosome of the same [54]. This imperative architecture of this locus implicates the need for ribosomal proteins for enhanced synthesis of essential proteins.

(b) PAIs of *Cellulosimicrobium cellulans* LMG 16121

The very first locus (LMG_PAI1) (Figure S3A) was completely dedicated to genes involved in cell wall biogenesis along with one integrase and transposase unit. These genes included UDP-glucose/GDP-mannose dehydrogenase, UDP-glucose pyrophosphorylase, dTDP-4-dehydrorhamnose reductase, dTDP-4-dehydrorhamnose 3,5-epimerase, capsular polysaccharide biosynthesis protein, glycosyltransferases and UDP-N-acetyl-D-mannosaminuronate dehydrogenase (Figure S3A). UDP-glucose associated enzymes along with glycosyltransferases forms a part of an operon responsible in host-cell recognition. Proteomic studies revealed that UDP-glucose pyrophosphorylase mutants showed reduced virulence in *Aeromonas hydrophila* implicating a direct link between these genes and pathogenicity [55]. These genes have also been illustrated to be involved in developing evasive strategies against killing mechanisms of the host by altering the cell surface [56].

Hypothetical proteins (n = 8) were annotated on the second locus (LMG_CPAI2) (Figure S3B) along with Type II secretion system, which is involved in enhancing both pathogenicity and environmental fitness. Two ORFs for transposases were also revealed along with putative membrane protein. Another very short PAI (LMG_CPAI3) (5 kbp)

was characterized by hypothetical proteins along with transcriptional activators (XRE family) and cell division protein (FtsK) (Figure S3C).

The mobility of PAIs can be phage-induced using helper proteins such as capsid proteins which help in packaging of the PAIs into transducing materials, which were discovered on LMG_CPAI4 (Figure S3D) [57]. Other phage-associated proteins identified included genes encoding phage-integrases (XerC and XerD family). Interestingly, genes encoding laminarinase and β-glucosidase-related glycosidases were also found on this locus, which are well known to be responsible for cellulolytic activity in bacteria. This can be directly linked to genus *Cellulosimicrobium* in which cellulose hydrolysis is one of the most notable characteristics [58]. Occurrence of these genes on flexible genome is of special interest as this implicates at lateral acquisition of cellulolytic trait by this taxon (Figure S3D). Two ORFs encoding F420-dependent oxidoreductase were also discovered on this locus which is already established to play a pivotal role in self-defense of pathogenic bacteria (specifically *Actinobacteria*) against oxidative stress [59]. This locus was also characterized by the presence of highest number of hypothetical proteins (n=13) (Figure S3D).

Fifth PAI (LMG_CPAI5) was marked by the presence of genes encoding arsenic resistance operon repressor and ClpX subunit (Figure S3E). D-alanyl-D-alanine carboxypeptidase was also revealed on this PAI, which is involved in cell wall biogenesis which is indicative of host-cell recognition [60]. Genes involved in repair mechanisms such as *ligD* were also found, this is reported to enhance the over-all fitness of the bacterial community. This locus also included *lsr2,* which is a histone-like bridging protein and is a well-known housekeeping gene, hence its occurrence on PAI implies at its relocation

(copy) from core-genome to the flexible gene pool as a fitness-enhancing determinant [60] (Figure S3E). Transcriptional regulators are often encoded on PAIs for regulation of virulent genes, hence modulating the pathogenicity [19]. This locus was characterized by different classes of TFs such as mycofactocin system transcriptional regulator, Cro-CI transcriptional regulators, and *cdaR* family transcriptional regulators. Cro-CI belong to a family of bacteriophage transcriptional regulators which are required for successful transcription of phage-related genes on the islands via regulatory networks such as phage capsid protein encoded on this specific locus [62]. *cdaR* is a common carbohydrate diacid regulon transcriptional regulator which was noted to be down-regulated as a result of compromised pathogenicity of *Salmonella enterica* serovar Typhimurium [20]. Gene encoding for beta-Keto acyl carrier protein reductase (BKR) was also detected on this locus, which is a key enzyme in rhamnolipid biosynthesis (Figure S3E) [22]. Rhamnolipids is a class of extracellular surfactants and recognized virulence factors, which are responsible for dissemination of surface signaling, biofilm formation and motility. Rhamnolipids are notorious virulence factors recovered from sputa of cystic fibrosis patients inflicted by *Pseudomonas aeruginosa*. ORF encoding for 17-β hydroxysteroid dehydrogenase-like protein was also predicted on this locus (Figure S3E), which forms a part of steroid based signaling network modulating (+/-) the signals between host and pathogen [63].

(c) PAIs of *Cellulosimicrobium cellulans* J36

The first annotated PAI locus (J36_CPAI1) in strain *C. cellulans* J36 revealed a relatively greater number of integrases and transposases accompanied with hypothetical proteins (Figure S4A). Additionally, hemin ABC transporter protein was also revealed on this

locus, implicating presence of an evolved iron-uptake system, which is required to regulate numerous virulence factors [64]. Copper binding protein along with copper export protein was also revealed again indicating presence of metallo-regulatory systems to enhance bacterial fitness. Anti-anti sigma factor was also annotated on this specific locus, which is involved in regulating virulent genes [65]. Antibiotic resistance genes were also revealed, including bleomycin resistance, doxorubicin resistance, and methyl viologen resistance protein. Phage-shock protein (psp) operon transcriptional activator was also annotated on this locus, which is established to maintain proton motive force (PMF) required for cellular motility by modulating flagellar movement [66].

The second locus (J36_CPAI2) (Figure S4B) was characterized by two ORFs encoding membrane proteins accompanied with three ORFs each for integrase and transposase. Hypothetical proteins were most abundant (n = 15) on this locus. Two ORFs for resolvase belonging to serine recombinase family were also annotated, which indicate at active HGT events via site-specific recombination (Figure S4B) [67]. The presence of a conjugal transfer protein (TrbC family) along with genes encoding for resolvase, integrase and transposase suggested that this island harbors a putative integrative conjugative element. In addition, this locus encoded regulators for arsenic resistance operon (n = 4) and flagellar biosynthesis (n = 1). Interestingly, mobilization proteins were also annotated which along with conjugal proteins are well-established contributors of evolution of bacterial pathogens via HGT of PAIs [68].

Dihydrofolate reductase and tetracyclin transcriptional regulator were annotated on third locus (J36_CPAI3) along with one ORF encoding transposase (Figure S4C). DEAD-box

helicase was also revealed which is established effector *fliC* gene responsible for flagellar driven motility using a deletion mutant assay [50].

Supplementary Figures

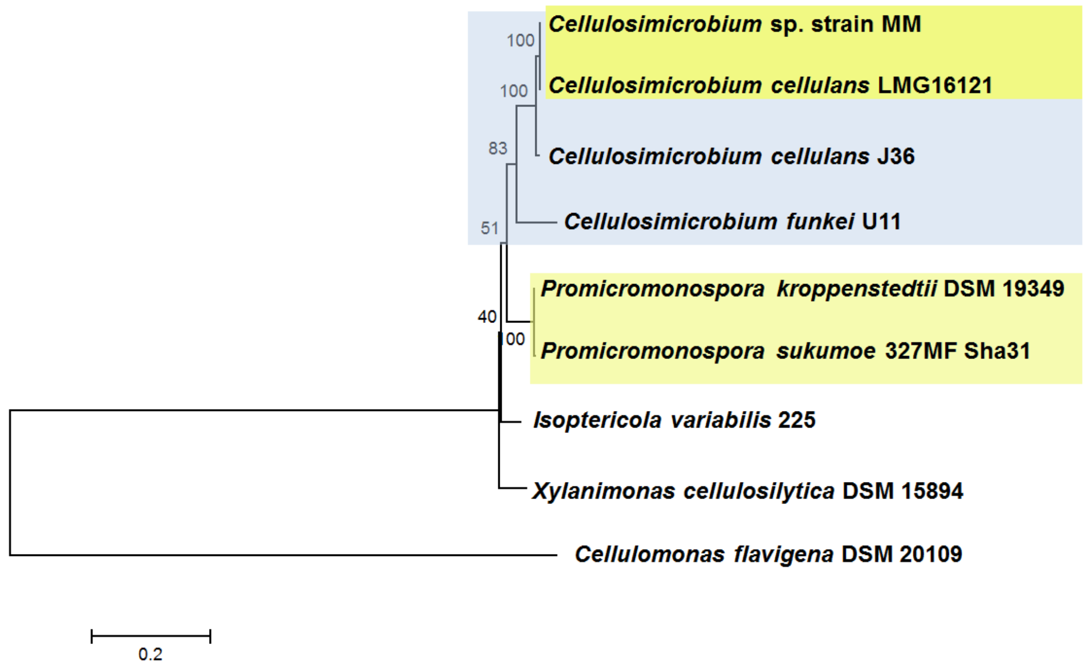

Figure S1: Rooted Maximum likelihood tree based on Jukes-Cantor model for family *Promicromonosporacae* using 400 bacterial marker genes with *Cellulosmonas flavigena* DSM 20109 as outgroup. The percentage of replicate trees in which the associated taxa clustered together in the bootstrap test (1000 replicates) are shown next to the branches. All the trees are drawn to scale, with branch lengths measured in the number of amino acid substitutions per site.

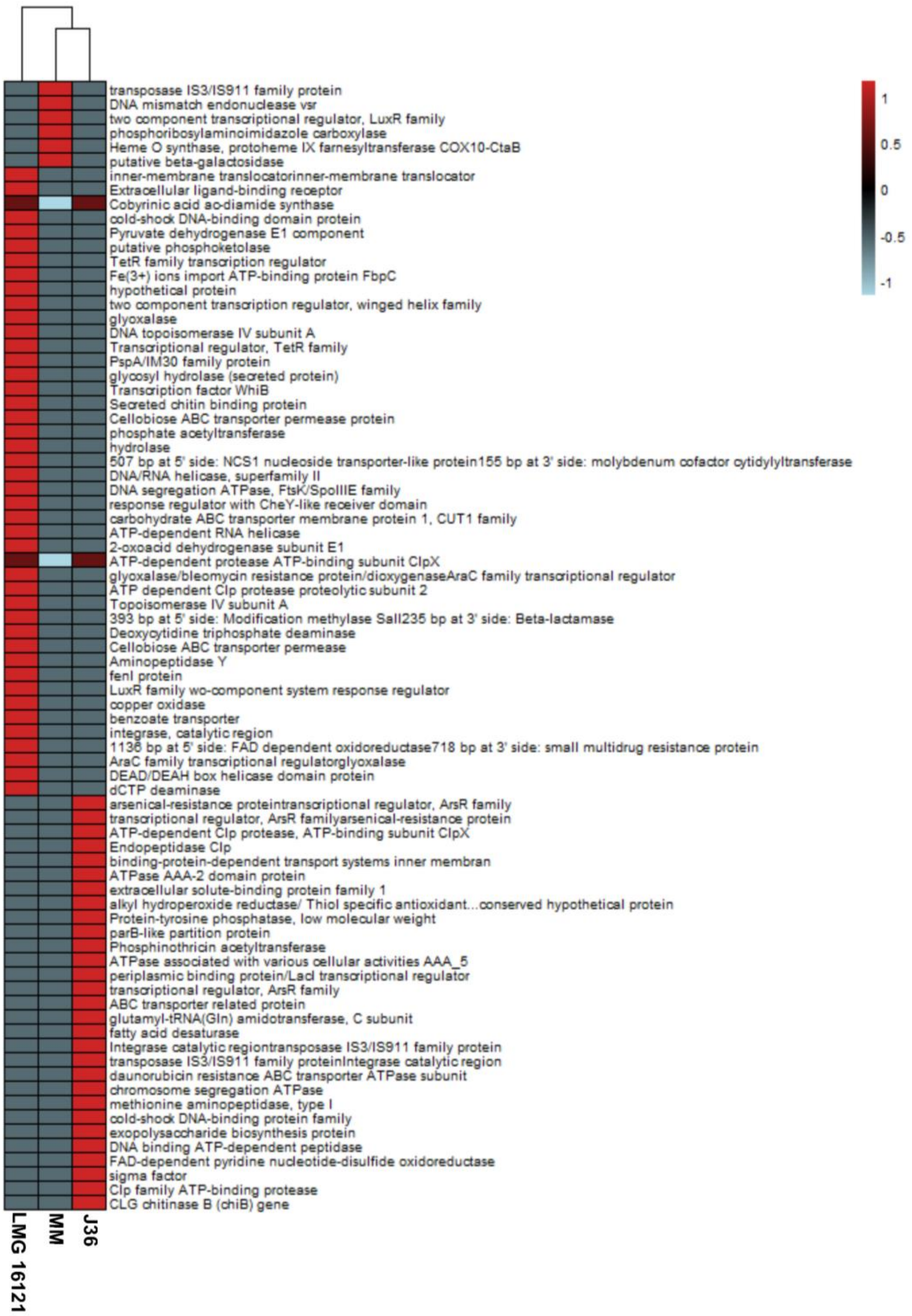

Figure S2: Heatmap with column dendrogram using hierarchical clustering based on HGT gene content using Euclidean distance matrix.

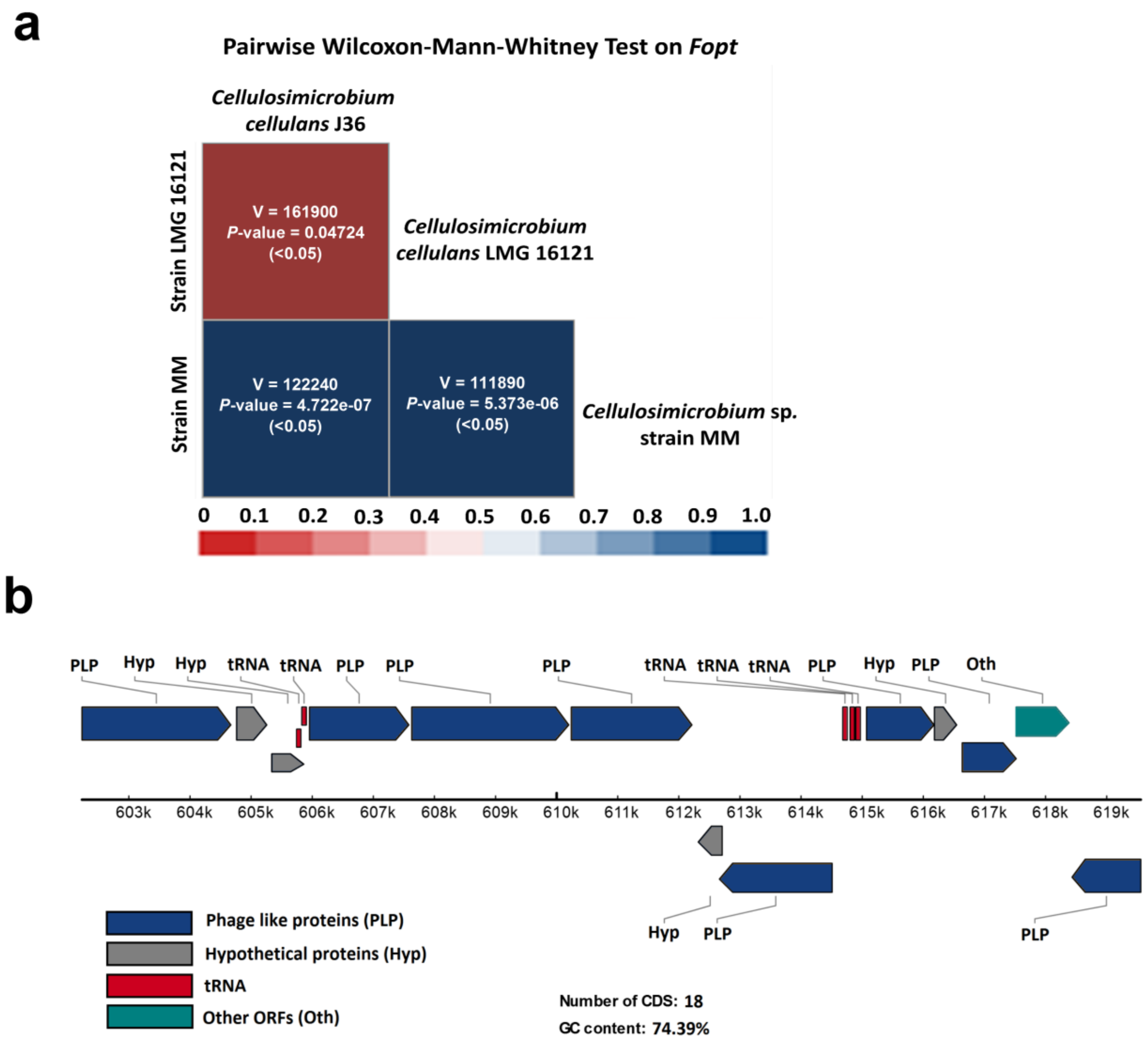

Figure S3: (a) The pairwise Wilcoxon ranked sum (V) and *P*-values across *Cellulosimicrobium* genomes (n = 3) with respect to Codon bias (F_{opt}) distribution using the Wilcoxon-Mann-Whitney test**. (b)** Schematic representation of phage-like region determined from the genome of strain MM.

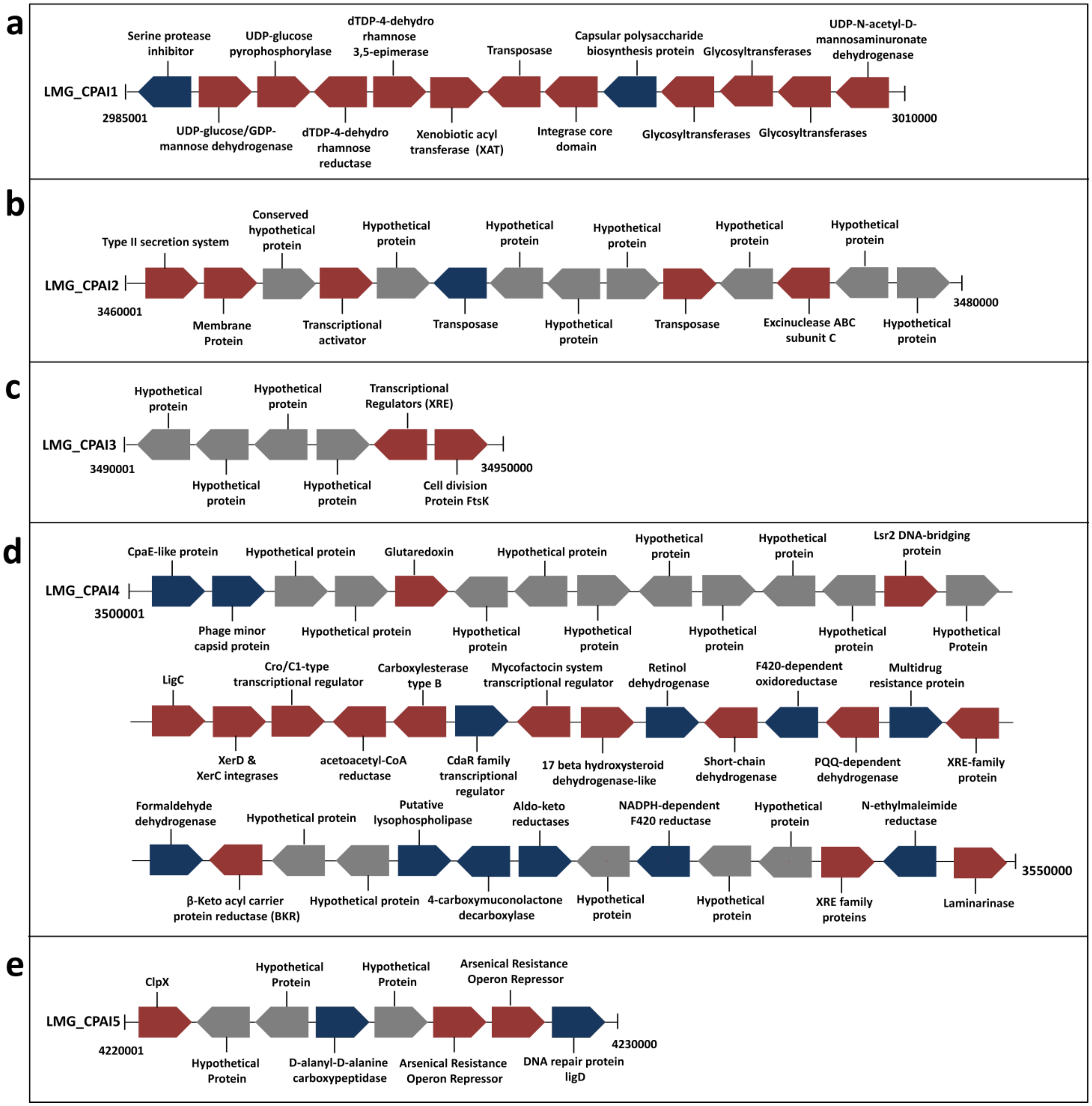

Figure S4: The schematic representation of ORFs annotated on five PAIs across genome of *Cellulosimicrobium cellulans* LMG16121. The direction of the ORFs shows the gene orientation. Nomenclature followed for each PAI belonging to strain LMG 16121 is

written as (a) LMG_CPAI1, (b) LMG_CPAI2, (c) LMG_CPAI3, (d) LMG_CPAI4, and (e) LMG_CPAI5 where “LMG” stands for the strain and “C” stands for the genus *Cellulosimicrobium*. Gray and red colored blocks represent non-virulent and virulent ORFs respectively. Slightly shaded colored blocks represent hypothetical proteins.

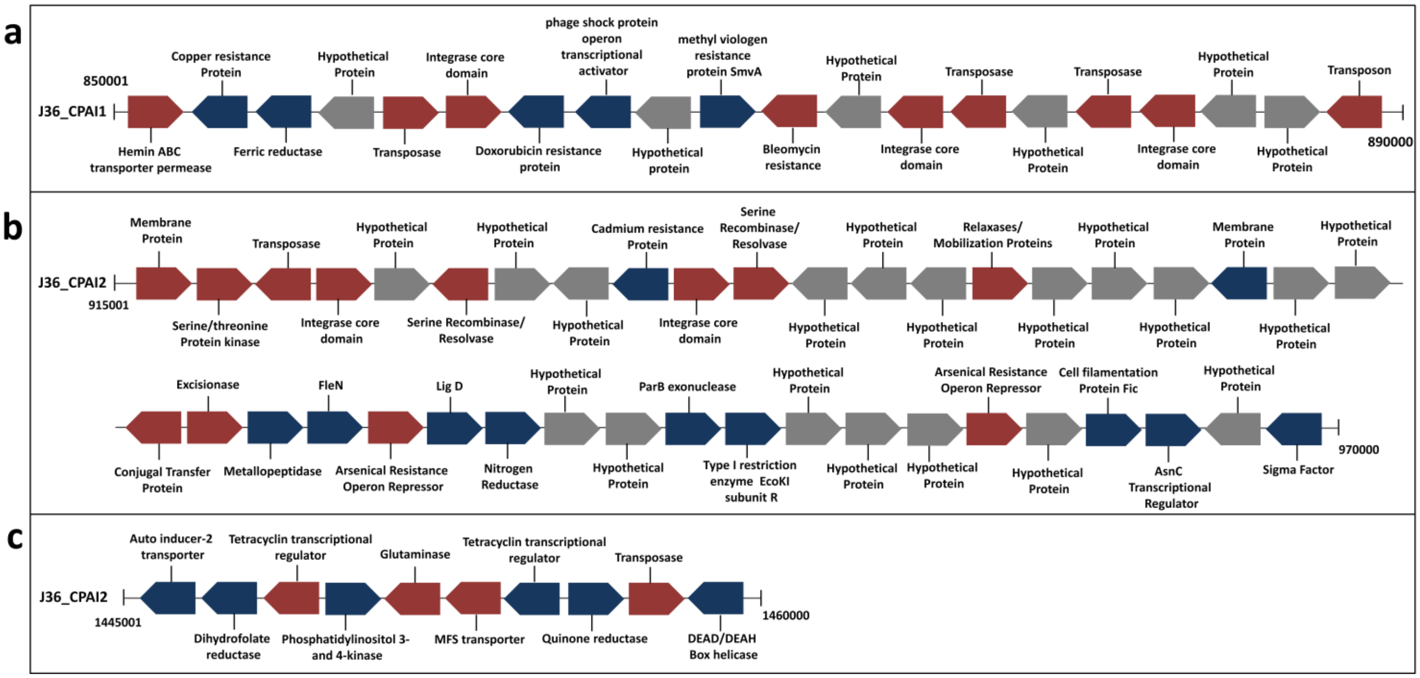

Figure S5: The schematic representation of ORFs annotated on 3 PAIs across genome of *Cellulosimicrobium cellulans* J36. The direction of the ORFs shows the gene orientation. A standard nomenclature was followed for each PAI belonging to strain J36 as (a) J36_CPAI1, (b) J36_CPAI2, and (c) J36_CPAI3 where “J36” stands for the strain and “C” stands for the genus *Cellulosimicrobium*. Grey and red colored blocks represent non-virulent and virulent ORFs respectively. Slightly shaded colored blocks represent hypothetical proteins.

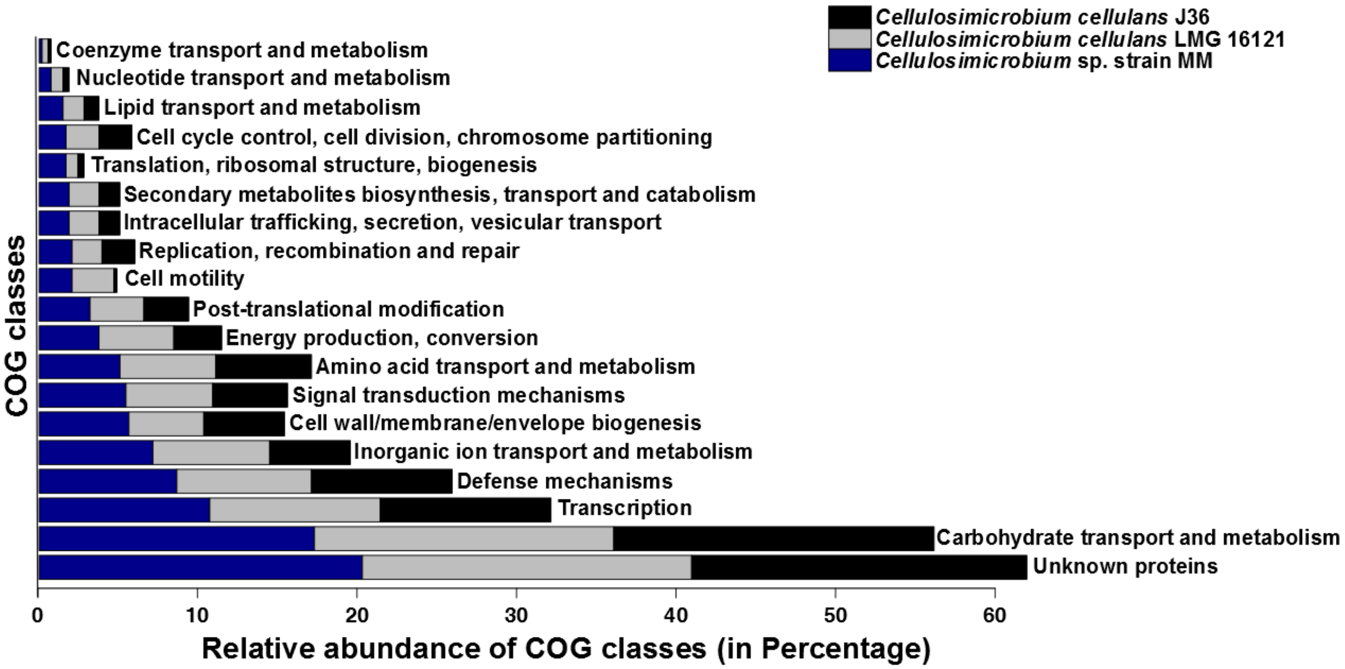

Figure S6: Distribution of COG classes (in percentage) across the pathogenic gene complements of the three genomes i.e. *Cellulosimicrobium* sp. strain MM, *Cellulosimicrobium cellulans* LMG 16121, and *Cellulosimicrobium cellulans* J36.

Supplementary Tables

Table S1: List of proteins annotated across PAIs from all *Cellulosimicrobium* ecotypes, established to be associated with human infections.

***Cellulosimicrobium* sp. strain MM**			
**PAI2**			
**S.No**	**ORFs Annotated on PAIs (BlastP)**	**Associated Human Pathogen**	**Reference**
**1.**	ClpP protease	*Staphylococcus aureus*	Michel *et al*., 2006 [1]
**2.**	TrwC relaxase	*Bartonella henselae*	Alperi *et al*., 2013 [2]
**3.**	VbhA anti-toxin	*Bartonella henselae*	Engel *et al*., 2012 [3]

**4.**	XerC recombinase	*Ureaplasma parvum*	Zimmerman *et al.*, 2013 [4]
		**PAI3**	
**5.**	Fluoroquinone resistance	Extraintestinal pathogenic *E. coli*	Guo *et al.*, 2013 [5]
**6.**	Sulfatase modifying factor	*Streptococcus pneumoniae*	McAllister *et al.*, 2012 [6]
		**PAI4**	
**7.**	PemK	*Clostridium botulinum*	Carter *et al.*, 2014 [7]
**8.**	ComEC	*Acinetobacter baumannii*	Wilharm *et al.*, 2013 [8]
**9.**	Phage portal protein	*Staphylococcus epidermidis*	Luan *et al.*, 2012 [9]
		**PAI5**	
**10.**	SecY	*Listeria monocytogenes*	Durack *et al.*, 2015 [10]
		***Cellulosimicrobium cellulans* LMG16121**	
		**PAI1**	
**11.**	UDP-glucose/GDP-mannose dehydrogenase	Group A *Streptococcus*	Cole *et al.*, 2012 [11]
**12.**	UDP-glucose pyrophosphorylase [Cell envelope biogenesis]	Group A *Streptococcus*	Cole *et al.*, 2012 [11]
**13.**	dTDP-4-dehydrorhamnose reductase [Cell envelope biogenesis]	Group A *Streptococcus*	Cole *et al.*, 2012 [11]
**14.**	dTDP-4-dehydrorhamnose 3,5-epimerase	Group A *Streptococcus*	Cole *et al.*, 2012 [11]
**15.**	Xenobiotic acyltransferase (XAT)	*Yersinia enterocolitica*	Garzetti *et al.*, 2012 [12]
		**PAI2**	
**16.**	Type II secretion system (T2SS)	uropathogenic *E. coli*	Kulkarni *et al.*, 2009 [13]
**17.**	Excinuclease ABC subunit C	*Leptospira interrogans*	Rakesh *et al.*, 2009 [14]

**PAI3**			
**18.**	FtsK like protein	*Mycobacterium tuberculosis*	Hett & Rubin, 2008 [15]
**PAI4**			
**19.**	Phage minor capsid protein 2	*Staphylococcus aureus*	Tallent *et al.*, 2007 [16]
**20.**	Lsr2	*Mycobacterium tuberculosis*	Bartek *et al.*, 2014 [17]
**21.**	Phage integrases	*Streptococcus agalactiae* serotype V	Tettelin *et al.*, 2002 [18]
**22.**	Cro/C1-like phage transcriptional regulator	*Staphylococcus aureus*	Ibarra *et al.*, 2013 [19]
**23.**	Putative CdaR family transcriptional regulator	*Salmonella enterica*	Lamichhane-Khadka *et al.*, 2011 [20]
**24.**	F420-dependent oxidoreductase	*Mycobacterium tuberculosis*	Gurumurthy *et al.*, 2013 [21]
**25.**	Beta-Keto acyl carrier protein reductase (BKR)	*Pseudomonas aeruginosa*	Miller *et al.*, 2006 [22]
**26.**	Putative lysophospholipase	*Pseudomonas aeruginosa*	Kovačić *et al.*, 2013 [23]
**PAI5**			
**27.**	ATP-dependent protease ATP-binding subunit ClpX	*Staphylococcus aureus*	McGillivray *et al.*, 2012 [24]
***Cellulosimicrobium cellulans* J36**			
**PAI1**			
**28.**	Hemin ABC transporter permease	*Corynebacterium diphtheria*	Bibb & Schmitt, 2010 [25]
**29.**	Copper resistance operon	*Listeria monocytogenes*	Corbett *et al.*, 2011 [26]
**30.**	Ferric reductase like transmembrane component	*Bordettella* spp.	Miethke and Marahiel, 2007 [27]

**31.**	Daunorubicin/doxorubicin resistance genes	*Pediococcus pentosaceus* and human cancer cell lines	Abuhammad and Zihlif, 2013 [28]
**32.**	Phage shock protein operon	*Yersinia enterocolitica*	Maxson and Darwin, 2006 [29]
**33.**	Methyl viologen resistance	*Salmonella typhimurium*	Hongo *et al.*, 1994 [30]
**34.**	Bleomycin resistance	*Staphylococcus aureus*	Gennimata *et al.*, 1996 [31]
**35.**	Arabinose operon control like protein	*Yersinia pestis*	Zhou *et al.*, 2004 [32]
		**PAI2**	
**36.**	Serine/threonine protein kinase	*Streptococcus* spp., *Mycobacterium, pseudomonas aeruginosa*	Pereira *et al.*, 2011 [33]
**37.**	Resolvase	*Salmonella enterica*	Merighi *et al.*, 2005 [34]
**38.**	Arsenical Resistance Operon Repressor	*Campylobacter jejuni*	Wang *et al.*, 2009 [35]
**39.**	Fe2+/Zn2+ uptake regulation proteins	*Escherichia coli, Shigella* spp., *Salmonella enterica*, *Klebsiella pneumoniae*	Porcheron *et al.*, 2013 [36]
**40.**	Relaxases/mobilisation proteins	*Legionella pneumophila*, *Brucella suis, Helicobacter pylori*	Alperi *et al.*, 2013 [2]
**41.**	Conjugal transfer protein, TrbC/VIRB2 family	*Bordettella pertussis, Helicobacter pylori*	Yeo and Waksman, 2004 [37]
**42.**	MerR family transcription regulator	*Streptococcus pneumoniae*	Potter *et al.*, 2010 [38]
**43.**	Antiactivator of flagellar biosynthesis FleN	*Pseudomonas aeruginos, Legionella pneumophila*	Jain and Kazmierczak, 2014 [39]
**44.**	ParB stage 0 sporulation protein J	*Mycobacterium leprae*	Fsihi *et al.*, 1996 [40]
**45.**	Cell filamentation protein Fic	*Mycobacterium tuberculosis*	Schmid *et al.*, 2006 [41]
		**PAI3**	

**46.**	Sporulation integral membrane protein	*Clostridium difficile*	Underwood *et al*., 2009 [42]
**47.**	Dihydrofolate reductase	*Mycobacterium tuberculosis*	Argyrou *et al*., 2006 [43]
**48.**	Tetracyclin transcriptional regulator	*Mycobacterium tuberculosis*	Anand *et al*., 2012 [44]
**49.**	Quinone reductase	*Helicobacter pylori, Vibrio cholera*	Kaakoush *et al*., 2007 [45]

Table S2: Pairwise ANI values between 8 genomes of family *Promicromonosporaceae.*

**Genomes**	***Cellulosimicrobium* sp. MM**	***Cellulosimicrobium cellulans* LMG1621**	***Cellulosimicrobium cellulans* J36**	***Cellulosimicrobium funkei* U11**	***Isoptericola variabilis* 225**	***Promicromonospora kroppenstedtii* DSM19349**	***Promicromonospora sukumoe* 327MFSha31**	***Xylanimonas cellulosilytica* DSM 15894**
Cellulosimicrobium sp. MM	100	98.23	88.24	85.29	81.17	80.28	79.71	79.88
Cellulosimicrobium cellulans LMG 1621	98.23	100	88.26	88.87	81.18	80.13	79.81	79.75
Cellulosimicrobium cellulans J36	88.24	88.26	100	88.87	81.22	80.35	79.93	79.72
Cellulosimicrobium funkei U11	85.29	88.87	88.87	100	80.98	80.23	79.85	79.59
Isoptericola variabilis 225	81.17	81.18	81.22	80.98	100	81.07	80.55	80.35
Promicromonospora kroppenstedtii DSM19349	80.28	80.13	80.35	80.23	81.07	100	94.52	80.01
Promicromonospora sukumoe 327MFSha31	79.71	79.81	79.93	79.85	80.55	94.52	100	79.67
Xylanimonas cellulosilytica DSM 15894	79.88	79.75	79.72	79.59	80.35	80.01	79.67	100

Table S3: Percent DNA-DNA hybridization (DDH) values calculated by Genome-Genome-Distance Calculator (GGDC) for pairwise combinations of *Cellulosimicrobium* sp. strain MM and other 7 genomes.

Query genome	Reference genome	DDH values
Cellulosimicrobium sp. strain MM	*C. cellulans* LMG 16121	75.6
Cellulosimicrobium sp. strain MM	*C. cellulans* J36	55.3
Cellulosimicrobium sp. strain MM	*C. funkei* U11	56
Cellulosimicrobium sp. strain MM	*Isoptericola variabilis* 225	20.9
Cellulosimicrobium sp. strain MM	*Promicromonospora kroppenstedtii* DSM19349	15.7
Cellulosimicrobium sp. strain MM	*Promicromonospora sukumoe* 327MFSha31	16
Cellulosimicrobium sp. strain MM	*Xylanimonas cellulosilytica* DSM15894	17.2

Supporting references

1. Michel, A. *et al*. Global regulatory impact of ClpP protease of Staphylococcus aureus on regulons involved in virulence, oxidative stress response, autolysis, and DNA repair. *J Bacteriol* **188**, 5783-96 (2006).
2. Alperi, A. *et al*. A Translocation Motif in Relaxase TrwC Specifically Affects Recruitment by Its Conjugative Type IV Secretion System. *J. Bacteriol*. **195**, 4999–5006 (2013).
3. Engel, P. *et al.* Adenylylation control by intra- or intermolecular active-site obstruction in Fic proteins. *Nature* **482**, 107–110 (2012).
4. Zimmerman, C.-U. R., Rosengarten, R. & Spergser, J. Interaction of the putative tyrosine recombinases RipX (UU145), XerC (UU222), and CodV (UU529) of Ureaplasma parvum serovar 3 with specific DNA. *FEMS Microbiol. Lett.* **340**, 55–64 (2013).
5. Guo, S. *et al*. Fluoroquinolone-resistant extraintestinal pathogenic Escherichia coli, including O25b-ST131, isolated from faeces of hospitalized dogs in an Australian veterinary referral centre. *J. Antimicrob. Chemother.* **68**, 1025-31 (2013).

6. McAllister, L. J., Ogunniyi, A. D., Stroeher, U. H. & Paton, J. C. Contribution of a Genomic Accessory Region Encoding a Putative Cellobiose Phosphotransferase System to Virulence of Streptococcus pneumoniae. *PLoS One* **7**, e32385; DOI: 10.1371/journal.pone.0032385 (2012).
7. Carter, A. T., Austin, J. W., Weedmark, K. A., Corbett, C. & Peck, M. W. Three Classes of Plasmid (47–63 kb) Carry the Type B Neurotoxin Gene Cluster of Group II Clostridium botulinum. *Genome Biol. Evol.* **6**, 2076–2087 (2014).
8. Wilharm, G., Piesker, J., Laue, M. & Skiebe, E. DNA Uptake by the Nosocomial Pathogen Acinetobacter baumannii Occurs during Movement along Wet Surfaces. *J. Bacteriol.* **195**, 4146–4153 (2013).
9. Luan, W. *et al*. Recombinant portal protein from Staphylococcus epidermidis bacteriophage CNPH82 is a 13-subunit oligomer. *Acta Crystallogr. Sect. F. Struct. Biol. Cryst. Commun.* **68**, 1267–1270 (2012).
10. Durack, J., Burke, T. P. & Portnoy, D. A. A prl mutation in SecY suppresses secretion and virulence defects of Listeria monocytogenes secA2 mutants. *J. Bacteriol*. **197**, 932–942 (2015).
11. Cole, J. N. *et al*. A Conserved UDP-Glucose Dehydrogenase Encoded outside the hasABC Operon Contributes to Capsule Biogenesis in Group A Streptococcus. *J. Bacteriol.* **194**, 6154–6161 (2012).
12. Garzetti, D., Bouabe, H., Heesemann, J. & Rakin, A. Tracing genomic variations in two highly virulent Yersinia enterocolitica strains with unequal ability to compete for host colonization. *BMC Genomics* **13**, 467; DOI: 10.1186/1471-2164-13-467 (2012).
13. Kulkarni, R. *et al*. Roles of putative type II secretion and type IV pilus systems in the virulence of uropathogenic Escherichia coli. *PLoS One* **4**, e4752; DOI: 10.1371/journal.pone.0004752 (2009).
14. Rakesh, S., Pradhan, D., & Umamaheswari, A. In silico approach for future development of subunit vaccines against Leptospira interrogans serovar Lai. *Int. J. Bioinformatics Res*. **1**, 85-92 (2009).
15. Hett, E. C. & Rubin, E. J. Bacterial Growth and Cell Division: a Mycobacterial Perspective. *Microbiol. Mol. Biol. Rev.* **72**, 126–156 (2008).
16. Tallent, S. M., Langston, T. B., Moran, R. G. & Christie, G. E. Transducing Particles of Staphylococcus aureus Pathogenicity Island SaPI1 Are Comprised of Helper Phage-Encoded Proteins. *J. Bacteriol.* **189**, 7520–7524 (2007).
17. Bartek, I. L. *et al*. Mycobacterium tuberculosis Lsr2 is a global transcriptional regulator required for adaptation to changing oxygen levels and virulence. *MBio* **5**, e01106–01114; DOI: 10.1128/mBio.01106-14 (2014).
18. Tettelin, H. *et al*. Complete genome sequence and comparative genomic analysis of an emerging human pathogen, serotype V Streptococcus agalactiae. *Proc. Natl. Acad. Sci. U.S.A.* **99**, 12391–12396 (2002).

19. Ibarra, J. A., Pérez-Rueda, E., Carroll, R. K. & Shaw, L. N. Global analysis of transcriptional regulators in Staphylococcus aureus. *BMC Genomics* **14**, 126; DOI: 10.1186/1471-2164-14-126 (2013).
20. Lamichhane-Khadka, R., Frye, J. G., Porwollik, S., McClelland, M. & Maier, R. J. Hydrogen-Stimulated Carbon Acquisition and Conservation in Salmonella enterica Serovar Typhimurium . *J. Bacteriol.* **193,** 5824–5832 (2011).
21. Gurumurthy, M. *et al*. A novel F420-dependent anti-oxidant mechanism protects Mycobacterium tuberculosis against oxidative stress and bactericidal agents. *Mol. Microbiol.* **87**, 744–755 (2013).
22. Miller, D. J., Zhang, Y.-M., Rock, C. O. & White, S. W. Structure of RhlG, an essential beta-ketoacyl reductase in the rhamnolipid biosynthetic pathway of Pseudomonas aeruginosa. *J. Biol. Chem.* **281**, 18025–18032 (2006).
23. Kovačić, F. *et al*. Structural and Functional Characterisation of TesA - A Novel Lysophospholipase A from Pseudomonas aeruginosa. *PLoS One* **8**, e69125; DOI: 10.1371/journal.pone.0069125 (2013).
24. McGillivray, S. M. *et al.* Pharmacological Inhibition of the ClpXP Protease Increases Bacterial Susceptibility to Host Cathelicidin Antimicrobial Peptides and Cell Envelope-Active Antibiotics. *Antimicrob. Agents Chemother.* **56**, 1854–1861 (2012).
25. Bibb, L. A. & Schmitt, M. P. The ABC transporter HrtAB confers resistance to hemin toxicity and is regulated in a hemin-dependent manner by the ChrAS two-component system in Corynebacterium diphtheriae. *J. Bacteriol.* **192,** 4606–4617 (2010).
26. Corbett, D. *et al*. The combined actions of the copper-responsive repressor CsoR and copper-metallochaperone CopZ modulate CopA-mediated copper efflux in the intracellular pathogen Listeria monocytogenes. *Mol. Microbiol.* **81**, 457–472 (2011).
27. Miethke, M. & Marahiel, M. A. Siderophore-Based Iron Acquisition and Pathogen Control. *Microbiol. Mol. Biol. Rev.* **71**, 413–451 (2007).
28. AbuHammad, S. & Zihlif, M. Gene expression alterations in doxorubicin resistant MCF7 breast cancer cell line. *Genomics* **101**, 213–220 (2013).
29. Maxson, M. E. & Darwin, A. J. Multiple promoters control expression of the Yersinia enterocolitica phage-shock-protein A (pspA) operon. *Microbiology* **152**, 1001–1010 (2006).
30. Hongo, E. *et al*. The methyl viologen-resistance-encoding gene smvA of Salmonella typhimurium. *Gene* **148**, 173–174 (1994).
31. Gennimata, D., Davies, J. & Tsiftsoglou, A. S. Bleomycin resistance in Staphylococcus aureus clinical isolates. *J. Antimicrob. Chemother*. **37,** 65–75 (1996).
32. Zhou, D. et al. Genetics of metabolic variations between Yersinia pestis biovars and the proposal of a new biovar, microtus. *J. Bacteriol*. **186**, 5147–5152 (2004).
33. Pereira, S. F. F., Goss, L. & Dworkin, J. Eukaryote-Like Serine/Threonine Kinases and Phosphatases in Bacteria. *Microbiol. Mol. Biol. Rev.* **75**, 192–212 (2011).

34. Merighi, M., Ellermeier, C. D., Slauch, J. M. & Gunn, J. S. Resolvase-in vivo expression technology analysis of the Salmonella enterica serovar Typhimurium PhoP and PmrA regulons in BALB/c mice. *J. Bacteriol.* **187**, 7407–7416 (2005).
35. Wang, L., Jeon, B., Sahin, O. & Zhang, Q. Identification of an Arsenic Resistance and Arsenic-Sensing System in Campylobacter jejuni. *Appl. Environ. Microbiol.* **75**, 5064–5073 (2009).
36. Porcheron, G., Garénaux, A., Proulx, J., Sabri, M. & Dozois, C. M. Iron, copper, zinc, and manganese transport and regulation in pathogenic Enterobacteria: correlations between strains, site of infection and the relative importance of the different metal transport systems for virulence. *Front. Cell. Infect. Microbiol.* **3**, 90; DOI: 10.3389/fcimb.2013.00090 (2013).
37. Yeo, H.-J. & Waksman, G. Unveiling Molecular Scaffolds of the Type IV Secretion System. *J. Bacteriol.* **186,** 1919–1926 (2004).
38. Potter, A. J., Kidd, S. P., McEwan, A. G. & Paton, J. C. The MerR/NmlR family transcription factor of Streptococcus pneumoniae responds to carbonyl stress and modulates hydrogen peroxide production. *J. Bacteriol.* **192**, 4063–4066 (2010).
39. Jain, R. & Kazmierczak, B. I. A conservative amino acid mutation in the master regulator FleQ renders Pseudomonas aeruginosa aflagellate. *PLoS One* **9**, e97439; DOI: 10.1371/journal.pone.0097439 (2014).
40. Fsihi, H. *et al*. Gene arrangement and organization in a approximately 76 kb fragment encompassing the oriC region of the chromosome of Mycobacterium leprae. *Microbiology* **142**, 3147–3161 (1996).
41. Schmid, M. C. *et al*. A translocated bacterial protein protects vascular endothelial cells from apoptosis. *PLoS Pathog*. **2**, e115; DOI: 10.1371/journal.ppat.0020115 (2006).
42. Underwood, S. *et al*. Characterization of the Sporulation Initiation Pathway of Clostridium difficile and Its Role in Toxin Production. *J. Bacteriol.* **191**, 7296–7305 (2009).
43. Argyrou, A., Vetting, M. W., Aladegbami, B. & Blanchard, J. S. Mycobacterium tuberculosis dihydrofolate reductase is a target for isoniazid. *Nat. Struct. Mol. Biol*. **13**, 408–413 (2006).
44. Anand, S. *et al*. Equilibrium binding and kinetic characterization of putative tetracycline repressor family transcription regulator Fad35R from Mycobacterium tuberculosis. *FEBS J.* **279**, 3214–3228 (2012).
45. Kaakoush, N. O., Kovach, Z. & Mendz, G. L. Potential role of thiol:disulfide oxidoreductases in the pathogenesis of Helicobacter pylori. *FEMS Immunol. Med. Microbiol*. **50,** 177–183 (2007).
46. Reynolds, T. B. Strategies for acquiring the phospholipid metabolite inositol in pathogenic bacteria, fungi and protozoa: making it and taking it. *Microbiology* **155**, 1386–1396 (2009).

47. Ollinger, J., O'Malley, T., Kesicki, E. A., Odingo, J. & Parish, T. Validation of the essential ClpP protease in Mycobacterium tuberculosis as a novel drug target. *J. Bacteriol.* **194**, 663–668 (2012).
48. Kjos, M., Salehian, Z., Nes, I. F. & Diep, D. B. An Extracellular Loop of the Mannose Phosphotransferase System Component IIC Is Responsible for Specific Targeting by Class IIa Bacteriocins. *J. Bacteriol.* **192**, 5906–5913 (2010).
49. Beceiro, A., Tomás, M. & Bou, G. Antimicrobial resistance and virulence: a successful or deleterious association in the bacterial world? *Clin. Microbiol. Rev*. **26**, 185–230 (2013).
50. Xu, Y. *et al*. An O Island 172 Encoded RNA Helicase Regulates the Motility of Escherichia coli O157:H7. *PLoS One* **8**, e64211; DOI: 10.1371/journal.pone.0064211 (2013).
51. Bukowski, M. *et al*. A regulatory role for Staphylococcus aureus toxin-antitoxin system PemIKSa. *Nat. Commun.* **4**, 2012; DOI: 10.1038/ncomms3012 (2013).
52. Smith, M. G. *et al*. New insights into Acinetobacter baumannii pathogenesis revealed by high-density pyrosequencing and transposon mutagenesis. *Genes Dev*. **21**, 601–614 (2007).
53. Brüssow, H., Canchaya, C. & Hardt, W.-D. Phages and the evolution of bacterial pathogens: from genomic rearrangements to lysogenic conversion. *Microbiol. Mol. Biol. Rev.* **68**, 560–602 (2004).
54. Fernández-Gómez, B. *et al*. Patterns and architecture of genomic islands in marine bacteria. *BMC Genomics* **13**, 347; DOI: 10.1186/1471-2164-13-347 (2012).
55. Vilches, S. *et al*. Mesophilic Aeromonas UDP-glucose pyrophosphorylase (GalU) mutants show two types of lipopolysaccharide structures and reduced virulence. *Microbiology* **153**, 2393–2404 (2007).
56. Ernst, R. K. *et al*. Specific lipopolysaccharide found in cystic fibrosis airway Pseudomonas aeruginosa. *Science* **286,** 1561–1565 (1999).
57. Quiles-Puchalt, N. *et al*. Staphylococcal pathogenicity island DNA packaging system involving cos-site packaging and phage-encoded HNH endonucleases. *Proc. Natl. Acad. Sci. U.S.A.* **111**, 6016–6021 (2014).
58. Ferrer, P. Revisiting the Cellulosimicrobium cellulans yeast-lytic β-1,3-glucanases toolbox: A review. *Microb. Cell Fact*. **5**, 10; DOI: 10.1186/1475-2859-5-10 (2006).
59. Bashiri, G., Rehan, A. M., Greenwood, D. R., Dickson, J. M. J. & Baker, E. N. Metabolic Engineering of Cofactor F 420 Production in Mycobacterium smegmatis. *PLoS One* **5**, e15803; DOI: 10.1371/journal.pone.0015803 (2010).
60. Kikuchi, H., Kim, S., Watanabe, K. & Watarai, M. Brucella abortusd-alanyl-d-alanine carboxypeptidase contributes to its intracellular replication and resistance against nitric oxide. *FEMS Microbiol. Lett*. **259**, 120–125 (2006).
61. Chen, J. M. *et al*. Roles of Lsr2 in colony morphology and biofilm formation of Mycobacterium smegmatis. *J. Bacteriol.* **188**, 633–641 (2006).

62. Schubert, R. A., Dodd, I. B., Egan, J. B. & Shearwin, K. E. Cro's role in the CI–Cro bistable switch is critical for λ's transition from lysogeny to lytic development. *Genes Dev*. **21**, 2461–2472 (2007).
63. García-Gómez, E. *et al.* Role of Sex Steroid Hormones in Bacterial-Host Interactions, Role of Sex Steroid Hormones in Bacterial-Host Interactions. *Biomed Res. Int*. **2013**, e928290; DOI: 10.1155/2013/928290 (2012).
64. Allen, C. E. & Schmitt, M. P. Novel Hemin Binding Domains in the Corynebacterium diphtheriae HtaA Protein Interact with Hemoglobin and Are Critical for Heme Iron Utilization by HtaA. *J. Bacteriol.* **193**, 5374–5385 (2011).
65. Kazmierczak, M. J., Wiedmann, M. & Boor, K. J. Alternative sigma factors and their roles in bacterial virulence. *Microbiol. Mol. Biol. Rev*. **69**, 527–543 (2005).
66. Jovanovic, G., Engl, C., Mayhew, A. J., Burrows, P. C. & Buck, M. Properties of the phage-shock-protein (Psp) regulatory complex that govern signal transduction and induction of the Psp response in Escherichia coli. *Microbiology* **156**, 2920–2932 (2010).
67. Ventura, M. *et al.* Genomics of Actinobacteria: tracing the evolutionary history of an ancient phylum. *Microbiol. Mol. Biol. Rev.* **71**, 495–548 (2007).
68. Ma, Z. *et al.* Insight into the specific virulence related genes and toxin-antitoxin virulent pathogenicity islands in swine streptococcosis pathogen Streptococcus equi ssp. zooepidemicus strain ATCC35246. *BMC Genomics* **14**, 377; DOI:10.1186/1471-2164-14-377 (2013).
